# Immunomodulatory Properties of DNA Hypomethylating Agents: Selecting the Optimal Epigenetic Partner for Cancer Immunotherapy

**DOI:** 10.3389/fphar.2018.01443

**Published:** 2018-12-07

**Authors:** Carolina Fazio, Alessia Covre, Ornella Cutaia, Maria Fortunata Lofiego, Patrizia Tunici, Carla Chiarucci, Sara Cannito, Gianluca Giacobini, James N. Lowder, Roberta Ferraldeschi, Pietro Taverna, Anna Maria Di Giacomo, Sandra Coral, Michele Maio

**Affiliations:** ^1^Department of Oncology, Center for Immuno-Oncology, Medical Oncology and Immunotherapy, University Hospital of Siena, Siena, Italy; ^2^Astex Pharmaceuticals, Inc., Pleasanton, CA, United States; ^3^Astex Therapeutics, Cambridge, United Kingdom

**Keywords:** DNA hypomethylating agent, epigenetics, cancer, immune phenotype, immunotherapy

## Abstract

DNA hypomethylating agents (DHAs) play a well-acknowledged role in potentiating the immunogenicity and the immune recognition of neoplastic cells. This immunomodulatory activity of DHAs is linked to their ability to induce or to up-regulate on neoplastic cells the expression of a variety of immune molecules that play a crucial role in host-tumor immune interactions. To further investigate the clinical potential of diverse epigenetic compounds when combined with immunotherapeutic strategies, we have now compared the tumor immunomodulatory properties of the first generation DHAs, azacytidine (AZA) and decitabine (DAC) and of the next generation DHA, guadecitabine. To this end, human melanoma and hematological cancer cells were treated *in vitro* with 1 μM guadecitabine, DAC or AZA and then studied by molecular and flow cytometry analyses for changes in their baseline expression of selected immune molecules involved in different mechanism(s) of immune recognition. Results demonstrated a stronger DNA hypomethylating activity of guadecitabine and DAC, compared to AZA that associated with stronger immunomodulatory activities. Indeed, the mRNA expression of cancer testis antigens, immune-checkpoint blocking molecules, immunostimulatory cytokines, involved in NK and T cell signaling and recruiting, and of genes involved in interferon pathway was higher after guadecitabine and DAC compared to AZA treatment. Moreover, a stronger up-regulation of the constitutive expression of HLA class I antigens and of Intercellular Adhesion Molecule-1 was observed with guadecitabine and DAC compared to AZA. Guadecitabine and DAC seem to represent the optimal combination partners to improve the therapeutic efficacy of immunotherapeutic agents in combination/sequencing clinical studies.

## Introduction

Epigenetic events are emerging as a hallmark of cancer development and progression, impairing immunogenicity and immune recognition of cancer cells, possibly favoring their escape from the host’s immune recognition (Sigalotti et al., 2005; Maio et al., 2015). One of the most widely studied epigenetic modifications in cancer is the aberrant methylation of DNA. It could occur through both global DNA hypomethylation, leading to genomic instability and possibly increasing the frequency of mutations and chromosomal abnormalities (Howard et al., 2008; Pogribny, 2010), and through the hypermethylation of specific genes leading to the impairment of the corresponding protein expression, mainly catalyzed by DNA methyltransferase (DNMT) enzymes (Sigalotti et al., 2014). The plasticity of epigenetic phenomena suggested the feasibility of their targeting by epigenetic drugs, such as DNA hypomethylating agents (DHAs), that can restore the physiologic epigenetic pattern by targeting DNMT enzymes (Maio et al., 2015). The most studied DHAs are nucleoside analogs of cytidine in which the cytosine ring has been modified to give them the DNMT inhibitory activity (Yoo and Jones, 2006). They include the first generation DHAs, azacytidine (AZA) and decitabine (DAC), FDA approved for the treatment of myelodysplastic syndromes (MDS) and acute myeloid leukemia (AML) (Saba, 2007; Cataldo et al., 2009), and the next generation DHA, guadecitabine. The latter is a dinucleotide of decitabine and deoxyguanosine designed to protect its active metabolite, DAC, from cytidine deaminase degradation resulting in a higher stability and a better tolerability of DAC in cancer patients (Yoo et al., 2007; Issa et al., 2015; Jueliger et al., 2016).

We have extensively demonstrated an epigenetic remodeling of cancer by DAC and guadecitabine as a result of the up-regulation and induction of different immune molecules and antigens involved in the immunogenicity and/or immune recognition of cancer cells of different histotype. Among them, HLA class I antigens, the co-stimulatory molecule ICAM-1, and tumor-associated antigens (TAA), such as the cancer testis antigens (CTAs) NY-ESO-1 and MAGE-A3 that are considered suitable therapeutic targets due to their high immunogenic potential (Coral et al., 1999, 2013). The functional role of these phenotypic changes is demonstrated by the significant improvement of tumor cells recognition by CTA-specific cytotoxic T lymphocytes (CTL) (Sigalotti et al., 2004) and by the induction of anti-CTA humoral immune response *in vivo* (Coral et al., 2006).

Besides an activity on genes directly involved in the recognition of tumor cell by T lymphocytes, transcriptional changes induced in tumors by DHAs affected also several genes involved in the viral defense pathway, leading to an “indirect” activation of anti-tumor immune response through the modulation of interferon (IFN) signaling (Chiappinelli et al., 2017).

Epigenetic remodeling has been also demonstrated to sensitize cancer cells to immune checkpoint (IC) blocking therapies, through the up-regulation of immunostimulatory cytokines [e.g., chemokine ligand 9 (CXCL9) and 10 (CXCL10)] that recruit T lymphocytes at tumor sites (Dunn and Rao, 2017), and/or through the up-regulation of the expression of IC molecules [i.e., cytotoxic T lymphocyte antigen 4 (CTLA-4), programmed death receptor 1 (PD-1) and its ligands (PD-L1 and PD-L2)] in MDS (Yang et al., 2014).

Innate immune cells play an important role in inhibiting cancer progression by complementing the effector activities of T cells; it has been demonstrated that these cells could exploit the action of epigenetic drugs by increasing tumor cell recognition and immune-mediated cell lysis. In this context, several studies reported that DAC-mediated hypomethylation could restore the NK group 2D ligands (NKG2DLs) [e.g., MHC class I–related chains (MIC) A and B] expression in tumors that represent an activating and a costimulatory signal for NK and T cells, respectively (Vasu et al., 2016; Zhang et al., 2016).

Although during the last years the pleiotropic immunomodulatory properties of different DHAs are consolidating, to the best of our knowledge no study investigated the differences among their activity. With the aim to optimize the therapeutic efficacy of DHAs in clinical setting and to identify the best epigenetic partner to be combined with cancer immunotherapy, we performed a comparative study of the immunomodulatory properties of the clinically approved DHAs (i.e., AZA and DAC) and of the next generation DHA guadecitabine, mainly focusing on the expression of different genes involved in different mechanism(s) of anti-tumor immunity.

## Materials and Methods

### Cell Lines

Human cutaneous melanoma cell lines (Mel 195, 275, 313, 346, 116, 120, 514, 142, 237, 403, 458, 345, 599, and 261) were generated from surgically removed metastatic lesions from melanoma patients, as previously described (Altomonte et al., 1993). Human hematological cancer cell lines (Daudi, HL-60, NALM-6, Raji, U-937, KG-1a, Jurkat, JY, Ri-1, K562) were purchased from American Type Culture Collection (Rockville, MD, United States).

Melanoma cells were grown in RPMI 1640 (Carlo Erba, Milan, Italy) supplemented with 10% heat-inactivated FBS (Biochrom, Berlin, Germany) and 2 mM L-glutamine (Biochrom, Berlin, Germany). Hematological tumor cell lines were grown in ISCOVE Basal Medium (Biochrom, Berlin, Germany) supplemented with 10% heat-inactivated FBS, 2 mM L-glutamine and 100 μg/μl penicillin/streptomycin (Biochrom, Berlin, Germany).

### Monoclonal Antibodies and Reagents

PE Mouse anti-human ICAM-1 clone 84H10 monoclonal antibody (mAb) was purchased from Beckman Coulter; alexafluor 488 mouse anti-human HLA class I clone W6/32 mAb was purchased from Biolegend; guadecitabine was kindly provided by Astex Pharmaceuticals, Inc. (Pleasanton, CA, United States); DAC was purchased from Abcam and AZA from Sigma Chemical Co.

### *In vitro* Tumor Cells Treatment With DHAs

Human melanoma (1 × 10^6^) and hematological cancer (1,2 × 10^6^) cell lines were seeded in T75 tissue culture flasks and treated 24 h later with 1 μM dose of guadecitabine or DAC (Coral et al., 2013), compared to an equimolar dose of AZA every 12 h for 2 days (4 pulses). At the end of the treatment (day 6th), cell lines were collected and analyzed. Control cultures were treated under similar experimental conditions without drugs.

### Quantitative Real-Time Methylation Specific PCR (qMSP) Analysis

Genomic DNA (500 ng) extracted from melanoma cell lines, using QIAmp DNA Blood mini Kit (Qiagen, Hilden, Germany), was subjected to modification with sodium bisulfite using the EZ DNA Methylation-Gold Kit (Zymo Research, CA, United States). Primers for the analysis of the methylation status of LINE-1 were designed using the free on-line software MethPrimer (Li and Dahiya, 2002), and are the follows: LINE-1 Unmethylated F: 5′-TGTGTGTGAGTTGAAGTAGGGT-3′, Unmethylated R: 5′-ACCCAATTTTCCAAATACAACCATCA-3′; LINE-1 Methylated F: 5′-CGCGAGTCGAAGTAGGGC-3′, Methylated R: 5′-ACCCGATTTTCCAAATACGACCG-3′. SYBR green qMSP reactions were performed with methylated- or unmethylated-specific primer pairs on 2 μl of bisulfite-modified genomic DNA. The copy number of methylated or unmethylated sequences for LINE-1 gene was established by extrapolation from the standard curves. The percentage (%) of methylation was defined as ratio between methylated molecules and the sum of methylated and unmethylated molecules and data were reported as % of LINE-1 demethylation ± standard deviation (SD) of treated vs. untreated cells. CpG Methyltransferase (New England BioLabs, Ipswich, MA, United States) and RepliG mini Kit (Qiagen, Hilden, Germany) were used to obtained positive (CTRL +) and negative (CTRL -) methylation control, respectively.

### Quantitative Real-Time RT-PCR Analysis

Total RNA was extracted by using Trizol reagent (Invitrogen, Milan, Italy) according to the manufacturer’s instruction. RNA extracted was digested with RNAse-free DNAse (Roche Diagnostics, Milan, Italy). Synthesis of cDNA was performed on 2 μg of total RNA using M-MLV reverse transcriptase (Invitrogen, Milan, Italy) and random hexamer primers (Promega, Milan, Italy), according to the manufacturer’s instructions. cDNA standards were obtained by RT-PCR amplification of the specific mRNAs and quantitated by NanoDrop2000 Spectrophotometer (Thermo Scientific, Massachusetts, United States). Quantitative real time RT-PCR were performed on 20 ng retrotranscribed total RNA in a final volume of 20 μl SYBR Green Master Mix (Applied Biosystems, Foster City, CA, United States) utilizing the 7500 Fast Real-Time PCR System (Applied Biosystems, Foster City, CA, United States) and software. The copy number of specific antigen and of the reference gene β-actin was established in each sample by extrapolation of the standard curve. The number of selected antigen cDNA molecules in each sample was then normalized to the number of cDNA molecules of β-actin. Gene expression was considered: (i) positive if numbers of gene/β-actin molecules were ≥ 1E-04; (ii) up-regulated if its positive expression was increased at least twice [Fold Change (FC) ≥2]. Data analyzed by multiparametric Dunn’s test with *p* < 0.05 were considered statistically significant. The primers used for the quantitative real-time RT-PCR analyses are listed in Supplementary Table 1.

### RT-PCR Analysis

RT-PCR reactions, using oligonucleotide primer sequences and PCR amplification programs specific for CTA family genes (i.e., MAGE-A2, -A4, -A10, GAGE1-2, SSX1-2, and SSX1-5), were performed as previously described (Sigalotti et al., 2004). The integrity of RNA and random primers-synthesized cDNA was confirmed by the amplification of all cDNA samples with β-actin-specific primers (Sigalotti et al., 2004). Five microliters of each RT-PCR sample were run on a 2% agarose gel, stained with green gel plus (Fisher Molecular Biology, Rome, Italy) and visualized by Gel doc XR (Bio-Rad Laboratories, Hercules, CA, United States). The primer sequences used for the quantitative RT-PCR are listed in Supplementary Table 2.

### Multi-Color Flow Cytometry

Cell surface expression of antigens on melanoma cell lines, treated and untreated with DHAs, was assessed by direct immunofluorescence staining followed by flow cytometry utilizing FACSCanto^TM^ (Beckman Coulter, Brea, CA, United States), according to the manufacturer’s instructions. Data were analyzed with the Kaluza software (Beckman Coulter, Brea, CA, United States). Results were expressed as % of positive cells and mean fluorescence intensity (MFI) values were subtracted from unstained cells values.

### Quantitative Relative Real-Time RT-PCR Analysis

Relative quantitative real time RT-PCR of Human Endogenous Retroviruses (HERV) Syncytin-1, -2, ERV9-1, ENV-MER34, ENV-Fb1, ENV-Fc2, ERV-FXA34, and ENV-T were performed on 20 ng retrotranscribed total RNA in a final volume of 20 μl SYBR Green Master Mix utilizing the 7500 Fast Real-Time PCR System and software. Interferon stimulated genes (ISG) were chosen from Taqman Gene Expression Assay (Applied Biosystem, Foster City, CA, United States): DDX58 (ID Hs01061436_m1), IFIT1 (ID Hs03027069_s1), IFIT2 (ID Hs01922738_s1), STAT1 (ID Hs01013996_m1), IFI27 (ID Hs01086373_g1), IFI6 (ID Hs00242571_m1), OAS1 (ID Hs00973637_m1), OAS2 (ID Hs00942643_m1), IRF7 (ID Hs01014809_g1), IRF9 (ID Hs00196051_m1), IFITM1 (ID Hs00705137_s1), IFITM3 (ID Hs03057129_s1), MX1 (ID Hs00895608_m1), MX2 (ID Hs01550811_m1), ISG15 (ID Hs01921425_s1), ISG20 (ID Hs00158122_m1), IFI44 (ID Hs00951349_m1), IFI44L (ID Hs00915292_m1), OASL (ID Hs00984387_m1), JAK1 (Hs01026983_m1), and JAK2 (Hs01078136_m1). The analyses were performed on 20 ng retrotranscribed total RNA in a final volume of 20 μl TaqMan Fast Advanced Master Mix (Applied Biosystem, Foster City, CA, United States) utilizing the 7500 Fast Real-Time PCR System and software. The 2^-ΔΔCT^ method was used to calculate relative expression levels. Results were expressed as FC of treated vs. untreated cells and gene expression was considered up-regulated when its positive expression was increased at least twice (FC ≥2).

## Results

### Comparative Analysis of the Demethylating Activity of Different DHAs in Human Cancer Cell Lines

To compare the demethylating activity of the different investigated DHAs in cancer cells, qMSP analysis was performed to measure the extent of LINE-1 methylation repetitive elements, chosen as a surrogate of the overall genomic DNA methylation, in 14 melanoma and in 10 hematological tumor cell lines treated with 1 μM guadecitabine, DAC or AZA.

**FIGURE 1:**
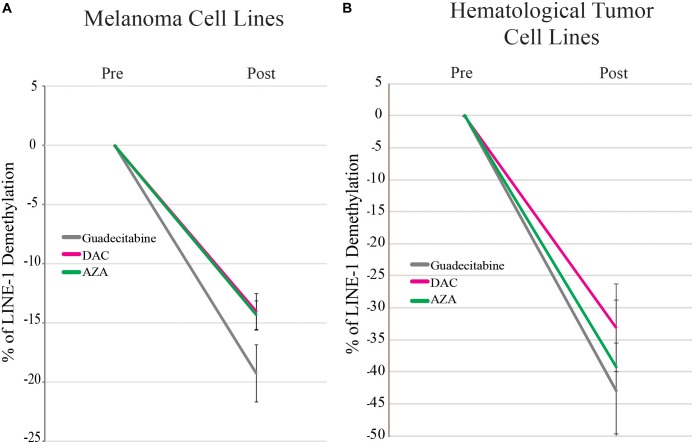
qMSP analysis of the methylation status of LINE-1 promoter in melanoma and hematological tumor cell lines treated with DHAs. Genomic DNA was extracted from 14 melanoma **(A)** and 10 hematological tumor **(B)** cell lines treated with 1 μM guadecitabine (gray), DAC (pink) or AZA (green). Real-time qMSP analyses of LINE-1 promoter were performed on bisulfite modified genomic DNA using methylated- or unmethylated-specific primer pairs. Data are reported as mean values ± SD of % of LINE-1 demethylation in DHAs-treated vs. untreated cells.

In melanoma and hematological tumor cell lines, the highest average global demethylation was observed after guadecitabine treatment. In detail, the mean of LINE-1 demethylation ± SD in guadecitabine- compared to DAC- and AZA-treated melanoma cells were: 19.2% (ranged from 40.5 to 1%) ± 12.6% vs. 14% (ranged from 36.1 to 0.3%) ± 10.9% and 14.3% (ranged from 35 to 0.6%) ± 8.5%, respectively (Figure 1A). In guadecitabine- vs. DAC- and AZA-treated hematological cancer cell lines, the mean of LINE-1 demethylation ± SD were: 43% (ranged from 67 to 13.4%) ± 17.3% vs. 33% (ranged from 60.8 to 7.2%) ± 20.6% and 39.2% (ranged from 72.6 to 8.4%) ± 26.7%, respectively (Figure 1B).

### Comparative Analysis of CTAs Expression in Human Cancer Cell Lines Treated With Different DHAs

**FIGURE 2:**
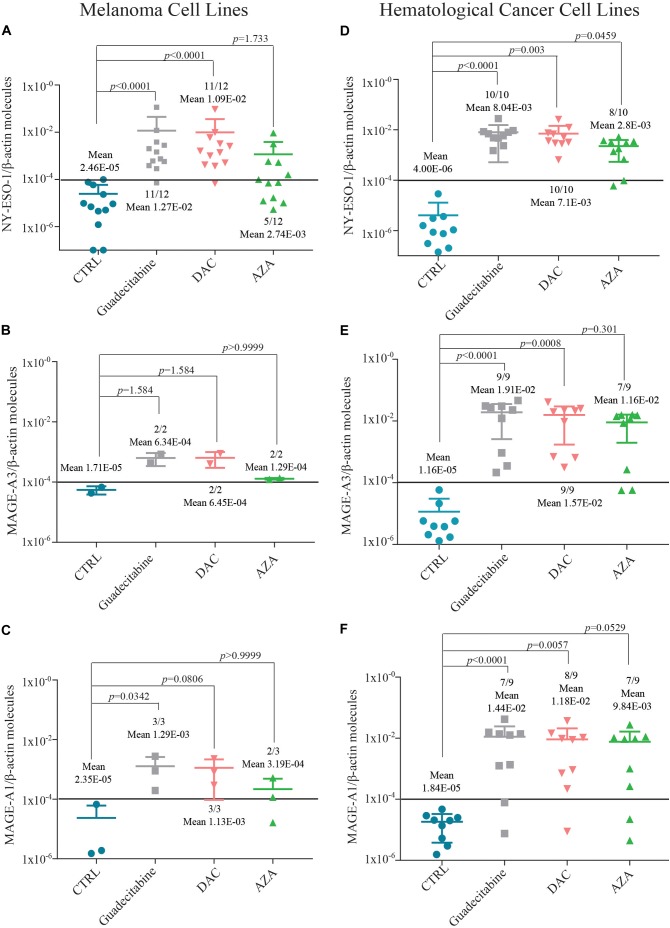
Quantitative RT-PCR analysis of CTAs expression in melanoma and hematological tumor cell lines treated with DHAs. Total RNA was extracted from CTA-negative melanoma and hematological tumor cell lines, either untreated (CTRL) or treated with 1 μM guadecitabine, DAC or AZA every 12 h for 2 days. Quantitative RT-PCR analyses were performed on retrotranscribed total RNA, utilizing NY-ESO-1-, MAGE-A3-, MAGE-A1- and β-actin-specific primers. CTAs expression was normalized to the expression of the β-actin gene. Scatter plots represent the number of NY-ESO-1, MAGE-A3 and -A1 molecules induced in melanoma **(A–C)** and hematological tumor **(D–F)** cells untreated and treated with investigated DHAs. Figures show also mean values ± SD of normalized CTAs molecules and *p* value calculated by Dunn’s test between DHAs-treated compared to untreated CTA-negative cells. Each data point represents individual cell line. Solid line (black) represents gene expression value ≥1E-04.

Quantitative real-time RT-PCR analyses were performed to investigate and compare levels of CTA (i.e., NY-ESO-1, MAGE-A3, and MAGE-A1) expression induced by the different DHAs treatments, in CTA-negative tumor cells selected among the investigated 14 human melanoma and 10 human hematological cancer cell lines. A *de novo* expression of NY-ESO-1, MAGE-A3 and -A1 was induced by guadecitabine or DAC treatment in 91.6% (11/12) (Figure 2A), 100% (2/2) (Figure 2B) and 100% (3/3) (Figure 2C) of CTA-negative melanoma cells, respectively. Conversely, a lower frequency of CTAs induction was observed following exposure to AZA resulting in a *de novo* expression of NY-ESO-1 and MAGE-A1 in 41.6% (5/12) (Figure 2A) and 66.6% (2/3) (Figure 2C) CTA-negative melanoma cells, respectively. The induction of MAGE-A3 was detected in 100% (2/2) of melanoma cells treated with AZA (Figure 2B). In addition, levels of CTAs expression, induced in melanoma cells, was stronger after treatment with guadecitabine or DAC vs. AZA, being the mean values of CTAs molecules ± SD 1.27E-02 ± 3.4E-02 and 1.09E-02 ± 2.73E-02 vs. 2.74E-03 ± 3.83E-03 for NY-ESO-1/β-actin (Figure 2A); 6.34E-04 ± 2.91E-04 and 6.45E-04 ± 3.47E-04 vs. 1.29E-04 ± 7.92E-06 for MAGE-A3/β-actin (Figure 2B); 1.29E-03 ± 1.33E-03 and 1.13E-03 ± 1.04E-03 vs. 3.19E-04 ± 2.88E-04 for MAGE-A1/β-actin (Figure 2C), respectively. Moreover, treatment with guadecitabine or DAC vs. AZA up-regulated (FC ≥2) the mRNA expression of NY-ESO-1 in 100% (2/2) and 100% (2/2) vs. 50% (1/2); of MAGE-A3 in 8.3% (1/12) and 16.7% (2/12) vs. 8.3% (1/12) and of MAGE-A1 in 27.3% (3/11) and 54.5% (6/11) vs. 36.3% (4/11) in CTA-positive melanoma cell lines (Supplementary Tables 3–5).

Small differences in the induction of CTAs expression by investigated DHAs were also observed in hematological cancer cell lines. In fact, treatment with guadecitabine or DAC vs. AZA induced the expression of NY-ESO-1 in 100% (10/10), 100% (10/10) vs. 80% (8/10) (Figure 2D), of MAGE-A3 in 100% (9/9), 100% (9/9) vs. 77.8% (7/9) (Figure 2E) and of MAGE-A1 in 77.8% (7/9), 88.9% (8/9) vs. 77.8% (7/9) (Figure 2F) CTA-negative hematologic cancer cell lines, respectively. No differences in the up-regulation (FC ≥2) of MAGE-A3 and MAGE-A1 were observed in CTA-positive hematological tumor cells among all investigated DHAs treatments (Supplementary Tables 7, 8).

Statistical analysis performed on data obtained from all (*n* = 14) investigated melanoma cells showed significant differences in levels of NY-ESO-1 (*p* < 0.0001) and MAGE-A1 (*p* < 0.05) expression detected after treatment with guadecitabine and DAC, but not with AZA, vs. untreated cells (Supplementary Tables 3, 5). No significant changes were observed for MAGE-A3 expression in all DHAs-treated melanoma cells (Supplementary Table 4). Data from all (*n* = 10) investigated hematological cancer cells showed significant (*p* < 0.05) differences in levels of NY-ESO-1 and MAGE-A1 expression after treatment with all DHAs, compared to untreated cells (Supplementary Tables 6, 8). Statistically significant (*p* < 0.005) differences in levels of MAGE-A3 expression were observed only in guadecitabine- and DAC-treated, compared to untreated hematological cancer cells (Supplementary Table 7).

The immunomodulatory activity of different DHAs on additional CTAs expression (i.e., MAGE-A2, -A4, -A10, GAGE1-2, SSX1-2, and SSX1-5) was investigated also by RT-PCR analysis in 14 melanoma and 10 hematological cancer cell lines treated with 1 μM guadecitabine, DAC or AZA. In melanoma cell lines, guadecitabine and DAC vs. AZA treatment induced the expression of MAGE-A2 in 100% (3/3) and 66.6% (2/3) vs. 33.3% (1/3); of MAGE-A4 in 66.6% (8/12) and 66.6% (8/12) vs. 25% (3/12); of MAGE-A10 in 42.8% (3/7) and 42.8% (3/7) vs. 42.8% (3/7); of GAGE1-2 in 100% (6/6) and 100% (6/6) vs. 83.3% (5/6); of SSX1-2 in 76.9% (10/13) and 76.9% (10/13) vs. 38.4% (5/13) and of SSX1-5 in 100% (10/10) and 100% (10/10) vs. 70% (7/10) (Table 1). Conversely, no differences in additional CTAs induction were observed among different DHAs treatments in hematological cancer cell lines (data not shown).

**Table 1:** RT-PCR analysis of CTAs expression in DHAs-treated melanoma cell lines^∗^.

	CTAs	MAGE-A2	MAGE-A4	MAGE-A10	GAGE1-2	SSX1-2	SSX1-5
Mel 346	CTRL	++^a^	-	-	+	-	-
	Guadecitabine	++	+	+	++	++	++
	DAC	++	+	+	++	+	++
	AZA	++	-	+	++	+	+
Mel 116	CTRL	++	-	++	-	-	-
	Guadecitabine	++	++	++	+	-	+
	DAC	++	++	++	+	-	++
	AZA	++	+	++	+	-	+
Mel 120	CTRL	++	-	+	+	-	+
	Guadecitabine	++	-	+	+	-	+
	DAC	++	-	+	+	-	+
	AZA	++	-	+	+	-	+
Mel 237	CTRL	++	-	++	++	-	+
	Guadecitabine	++	-	++	++	-	++
	DAC	++	-	++	++	-	++
	AZA	++	-	++	++	-	+
Mel 403	CTRL	++	-	+	-	-	+
	Guadecitabine	++	-	+	++	+	+
	DAC	++	-	+	++	+	+
	AZA	++	-	+	+	+	+
Mel 313	CTRL	-	-	-	-	-	-
	Guadecitabine	+	+	-	++	+	++
	DAC	-	+	-	++	+	++
	AZA	-	-	-	+	-	-
Mel 195	CTRL	-	-	-	-	-	-
	Guadecitabine	++	++	-	++	+	++
	DAC	++	++	-	++	+	++
	AZA	-	-	-	-	+	-
Mel 275	CTRL	++	-	++	-	-	-
	Guadecitabine	++	++	++	++	+	++
	DAC	++	++	++	++	+	++
	AZA	++	+	++	++	+	++
Mel 458	CTRL	-	-	-	++	-	-
	Guadecitabine	+	+	-	++	+	++
	DAC	+	+	-	++	+	++
	AZA	+	-	-	++	-	+
Mel 599	CTRL	+	-	-	-	-	-
	Guadecitabine	+	-	-	++	+	++
	DAC	+	-	-	++	+	+
	AZA	+	-	-	++	-	+
Mel 261	CTRL	+	-	-	+	-	-
	Guadecitabine	++	+	+	++	+	++
	DAC	++	+	+	++	+	++
	AZA	+	-	+	++	-	-
Mel 514	CTRL	++	-	++	++	-	-
	Guadecitabine	++	++	++	++	+	+
	DAC	++	++	++	++	+	+
	AZA	++	++	++	++	-	+
Mel 345	CTRL	++	++	-	+	-	-
	Guadecitabine	++	++	+	++	+	++
	DAC	++	++	+	++	+	++
	AZA	++	++	+	++	+	++
Mel 142	CTRL	++	++	++	++	++	++
	Guadecitabine	++	++	++	++	++	++
	DAC	++	++	++	++	++	++
	AZA	++	++	++	++	++	++


*^∗^RT-PCR analyses were performed on total RNA extracted from 14 melanoma cell lines either untreated (CTRL) or treated with 1μM guadecitabine, DAC or AZA.*

*^*a*^Intensity of RT-PCR products: -, not detectable; +, weak; ++, strong.*

### Comparative Analysis of HLA Class I Antigens and Co-stimulatory Molecules Expression in Human Cancer Cell Lines Treated With Different DHAs

The immunomodulatory activity of different DHAs on the constitutive expression levels of HLA class I antigens and the co-stimulatory molecule, ICAM-1, was evaluated in 14 melanoma cell lines and 10 hematological cancer cell lines treated with 1 μM guadecitabine, DAC or AZA, by flow cytometry.

Results showed that treatment with DHAs modulated the constitutive expression of both antigens in all investigated melanoma and hematological cancer cell lines. In detail, in untreated vs. guadecitabine-, DAC- or AZA-treated melanoma cells, mean values of MFI ± SD of HLA class I antigens were 100.60 ± 103.15 vs. 153.40 ± 130.17, 155.80 ± 126.63 and 116.30 ± 107.67 and mean values of MFI ± SD of ICAM-1 were 45.80 ± 37.81 vs. 64.50 ± 38.94, 64.8 ± 45.25 and 45.70 ± 36.56 (Table 2). In hematological cancer cell lines mean values of MFI ± SD of HLA class I antigens were 116.80 ± 117.90 vs. 311.80 ± 243.50, 313.30 ± 248.30 and 215.50 ± 182.40 and mean values of MFI ± SD of ICAM-1 molecules were 64.10 ± 70.60 vs. 202.90 ± 253.20, 205.80 ± 243.70 and 158.40 ± 196.20, in untreated vs. guadecitabine-, DAC- or AZA-treated cells, respectively (Table 3).

**Table 2:** MFI by flow cytometry analysis of DHAs-treated melanoma cell lines^∗^.

	HLA class I^a^				ICAM-1^b^			
		
	CTRL	Guadecitabine	DAC	AZA	CTRL	Guadecitabine	DAC	AZA
Mel 195	10.60	41.47	52.38	23.58	35.22	91.05	115.34	69.45
Mel 313	145.62	300.27	328.83	244.41	12.71	26.89	33.14	15.23
Mel 275	39.01	106.24	110.23	87.02	139.59	118.91	171.77	138.96
Mel 346	64.50	76.60	73.80	44.90	15.4	16.00	17.60	17.70
Mel 116	151.93	245.77	267.44	173.57	54.25	87.98	93.35	75.95
Mel 120	35.20	54.40	50.60	43.80	48.10	51.70	49.20	48.80
Mel 514	408.75	471.22	425.21	405.77	50.99	66.30	63.17	46.57
Mel 142	62.07	66.48	74.43	51.95	31.21	73.20	49.75	12.03
Mel 237	33.47	40.50	33.39	27.87	33.20	28.95	32.16	39.29
Mel 403	77.28	144.77	138.48	125.06	18.96	29.84	30.55	20.76
Mel 458	47.04	90.45	97.17	53.46	15.35	24.75	26.40	10.00
Mel 345	77.38	129.51	130.41	103.22	46.55	80.97	70.10	38.54
Mel 599	57.77	64.94	70.58	47.26	23.09	24.11	32.52	20.13
Mel 261	197.84	314.62	328.04	196.78	166.63	139.78	122.40	86.94
Mean	100.60	153.40	155.80	116.30	45.80	61.50	64.80	45.70
SD	103.15	130.17	126.63	107.67	37.81	38.94	45.25	36.56
Dunn Test vs. CTRL		*p* = 0.0004	*p <* 0.0001	*p >* 0.9999		*p* = 0.0252	*p* = 0.0013	*p >* 0.9999


***^∗^**Melanoma cell lines untreated (CTRL) and treated with 1μM guadecitabine, DAC or AZA were incubated with anti-human HLA class I (^*a*^) and anti-ICAM-1 (^*b*^) mAbs and studied by flow cytometry. Data were analyzed by using Kaluza software. Mean fluorescence intensity (MFI) of cell surface staining.*

**Table 3:** MFI by flow cytometry analysis of DHAs-treated hematological tumor cell lines^∗^.

	HLA class I^a^				ICAM-1^b^			
		
	CTRL	Guadecitabine	DAC	AZA	CTRL	Guadecitabine	DAC	AZA
JY	323.50	601.77	670.23	539.93	152.88	193.07	235.77	149.21
KG-1a	166.12	331	350.79	365.28	18.28	48.11	45.55	23.31
Ri-1	50.62	602.54	624.65	393.37	129.28	606.86	567.30	452.94
NALM-6	43.98	502.20	475.70	300.94	114.70	602.38	576.41	475.26
Rajy	41.31	542.19	482.72	116.49	177.39	459.20	483.72	369.83
U-937	245.40	313.10	293.44	244.43	11.33	27.08	25.44	25.76
JURKAT	48.53	71.92	76.50	62.80	0.51	0.96	1.08	0.58
K562	2.17	5.75	7.36	4.26	8.96	7.54	8.76	5.05
HL-60	246.28	146.90	151.31	127.59	1.06	5.92	6.57	3.63
Daudi	0.12	0.34	0.24	0.00	26.25	77.58	107.66	78.74
Mean	116.80	31.80	313.30	215.50	64.10	202.90	205.80	158.40
SD	117.97	243.52	248.36	182.39	70.63	253.18	243.68	196.22
Dunn Test vs. CTRL		*p* = 0.0097	*p* = 0.003	*p >* 0.9999		*p* = 0.0055	*p* = 0.0016	*p* = 0.8961


***^∗^**Hematological tumor cell lines untreated (CTRL) and treated with 1 μM guadecitabine, DAC or AZA were incubated with anti-human HLA class I (^*a*^) and anti-ICAM-1 (^*b*^) mAbs and studied by flow cytometry. Data were analyzed by using Kaluza software. Mean Fluorescence intensity (MFI) of cell surface staining.ii.*

Statistical analysis performed on data obtained from all investigated 14 melanoma and 10 hematological cancer cells showed significant (*p* < 0.05) differences in MFI of HLA class I and ICAM-1 expression detected after treatment with guadecitabine and DAC, but not with AZA, vs. untreated cells (Tables 2, 3).

No differences in the % of HLA class I antigens and ICAM-1 positive cells were observed after DHAs treatment, in all investigated melanoma and hematological tumor cell lines (data not shown).

### Comparative Analysis of IC Molecules Expression in Human Cancer Cell Lines Treated With Different DHAs

**FIGURE 3:**
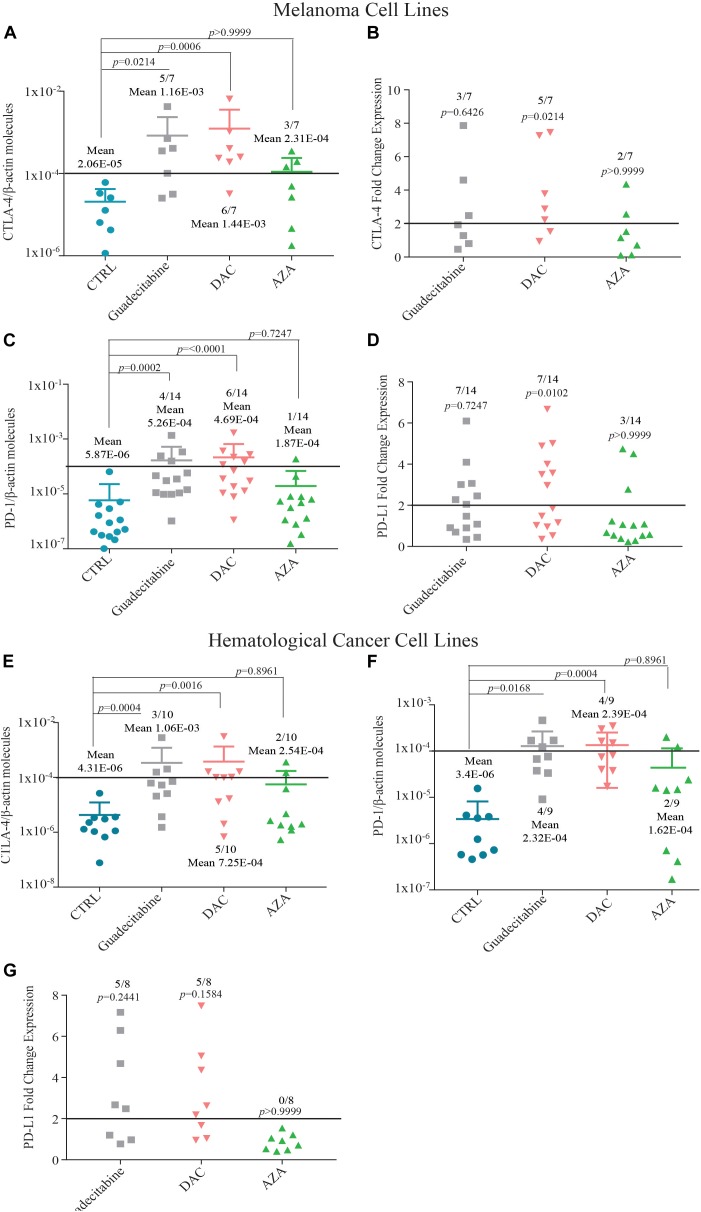
Quantitative RT-PCR analysis of IC molecules expression in melanoma and hematological cancer cell lines treated with DHAs. Total RNA was extracted from melanoma **(A–D)** and hematological cancer **(E,G)** cell lines, untreated (CTRL) or treated with 1 μM guadecitabine, DAC or AZA. Quantitative real-time RT-PCR analyses were performed on retrotranscribed total RNA, utilizing CTLA-4-, PD-1-, PD-L1- and β-actin-specific primers. ICs expression was normalized to the expression of the β-actin gene. Scatter plots represent: (i) the number of IC molecules in untreated and DHAs-treated IC-negative melanoma **(A,C)** and hematological cancer **(E,F)** cells; (ii) the FC of IC expression in untreated vs. DHAs-treated IC-positive melanoma **(B,D)** and hematological cancer **(G)** cells. Figures show also mean values ± SD of normalized ICs molecules **(A,C,E,F)** and *p*-value calculated by Dunn’s test between mean values of IC expression in DHAs-treated compared to untreated cells. Each data point represents individual cell line. Solid line (black) represents gene expression value ≥1E-04 or FC expression value ≥2.

Quantitative real-time RT-PCR analyses were performed to study the effects of treatment with different DHAs on the expression levels of IC (i.e., CTLA-4, PD-1 and PD-L1) mRNA molecules, in IC-negative cancer cells, selected among the investigated 14 melanoma and 10 hematological cancer cell lines. A *de novo* expression of CTLA-4 was induced in 71.4% (5/7) and 85.7% (6/7) vs. 42.8% (3/7) IC-negative melanoma cells, treated with guadecitabine and DAC vs. AZA, respectively, with mean values ± SD of CTLA-4 molecules 1.16E-03 ± 1.73E-03 and 1.44E-03 ± 2.49E-03 vs. 2.31E-04 ± 1.07E-04, respectively (Figure 3A). Consistent with a different immunomodulatory activity between investigated DHAs, treatment of CTLA-4-positive melanoma cells with guadecitabine, DAC or AZA up-regulated (FC ≥2) the constitutive expression levels of CTLA-4 in 42.8% (3/7), 71.4% (5/7) and 28.6% (2/7) melanoma cell lines, respectively (Figure 3B). A *de novo* induction of PD-1 was detected in 28.5% (4/14) and 42.8% (6/14) vs. 7.1% (1/14) melanoma cells treated with guadecitabine or DAC vs. AZA, respectively, with no differences in the mean values of PD-1 molecules induced by all DHAs treatments (Figure 3C).

A positive expression of PD-L1-specific mRNA was detected in all investigated melanoma cells, and it was up-regulated (FC ≥ 2) with a higher frequency in melanoma cells treated with guadecitabine (50%, 7/14) or DAC (50%, 7/14), compared to AZA (21.4%, 3/14) (Figure 3D).

A similar trend of IC modulation by DHAs was observed in hematological cancer cell lines. The expression of CTLA-4-specific mRNA was induced in 30% (3/10), 50% (5/10), and 20% (2/10) of CTLA-4-negative hematological cancer cells treated with guadecitabine, DAC or AZA, respectively. This induction was stronger after treatment with guadecitabine or DAC vs. AZA, being the mean values of CTLA-4 molecules ± SD 1.06E-03 ± 1.53E-03, 7.25E-04 ± 1.33E-03 vs. 2.54E-04 ± 1.48E-04, respectively (Figure 3E). Likewise, a higher frequency of PD-1 induction was detected in PD-1-negative hematological cancer cells after treatment with both guadecitabine or DAC (44.4%, 4/9) compared to AZA treatment (22.2%, 2/9) with no differences in the mean values of PD-1 molecules induced by all DHAs treatments (Figure 3F).

Moreover, treatment with both guadecitabine or DAC up-regulated (FC ≥2) constitutive levels of PD-L1 expression in 62.5% (5/8) positive hematological cancer cells, compared to 0% in AZA-treated cells (Figure 3G). Induction of PD-L1 was observed in one PD-L1-negative cell line only after DAC treatment (Supplementary Table 14).

Statistical analysis performed on data obtained from all investigated melanoma and hematological cancer cells showed significant (*p* < 0.05) differences in levels of CTLA-4 and PD-1 expression detected after guadecitabine and DAC treatment, but not after AZA, compared to untreated cells (Supplementary Tables 9, 10, 12, 13). Moreover, levels of PD-L1 expression observed only after DAC treatment were significantly different (*p* < 0.05) in both melanoma and hematological cancer cells vs. untreated cells (Supplementary Tables 11, 14).

### Comparative Analysis of the Activity of Different DHAs in TME Immunomodulation

The study of the immunomodulatory effects of different DHAs was expanded by qRT-PCR analysis of changes in the expression of selected immunostimulatory molecules (e.g., CXCL10 and CXCL9, MICA and MICB) in melanoma (*n* = 14) and hematological tumor (*n* = 10) cell lines treated with 1 μM guadecitabine, DAC or AZA.

Treatment with guadecitabine or DAC vs. AZA induced the mRNA expression of CXCL10 in 38.5% (5/13) and 30.7% (4/13) vs. 15.4% (2/13) chemokine-negative melanoma cell lines, with no differences in the mean values of induced CXCL10 molecules among investigated DHAs (Supplementary Table 15). The expression of CXCL9 was induced only by DAC treatment in 20% (2/10) chemokine-negative melanoma cells, while it was up-regulated (FC ≥2) by treatment with guadecitabine and DAC in 25% (1/4) and 75% (3/4) chemokine-positive melanoma cells, respectively, compared to 0% in AZA-treated cells (Supplementary Table 16).

Also the NK activating ligand MICB was induced/up-regulated by guadecitabine and DAC treatment in 57.1% (8/14) and by AZA in 14.2% (2/14), while MICA was up-regulated (FC ≥2) by guadecitabine and DAC treatment in 14.2% (2/14) and by AZA in 7.1% (1/14) of melanoma cell lines (Supplementary Tables 17, 18).

Similarly, in hematological tumor cell lines, treatment with guadecitabine or DAC vs. AZA induced the mRNA expression of CXCL10 in 50% (3/6), 66.6% (4/6) vs. 16.6% (1/6) of chemokine-negative cells (Supplementary Table 19), with a mean values ± SD of induced CXCL10 molecules of 2.41E-02 ± 2.95E-02 and 2.52E-02 ± 4.19E-02 vs. 4.50E-03 ± 4.50E-03, respectively; while the expression of CXCL9 was induced in 75% (6/8), 75% (6/8) vs. 25% (2/8) of guadecitabine- or DAC- vs. AZA-treated cells, respectively (Supplementary Table 20), with no differences in the mean values of CXCL9 molecules induced by all DHAs treatments.

No differences were observed in the number of positive hematological tumor cell lines in which CXCL10, CXCL9, MICA and MICB expression was up-regulated (FC ≥2) by all investigated DHAs treatments (Supplementary Tables 19–22).

Statistical analysis performed on data obtained from all investigated melanoma cells showed significant differences (*p* < 0.05) in levels of expression of all immunostimulatory molecules after guadecitabine and DAC treatment (Supplementary Tables 13–16), but not after AZA exposure, compared to untreated cells.

Conversely, data from all hematological cancer cells showed significant (*p* < 0.005) differences in levels of CXCL9 and MICA expression only after guadecitabine and DAC treatment vs. untreated cells; while significant (*p* < 0.05) differences in levels of CXCL10 and MICB expression were observed only after AZA treatment, compared to untreated cells (Supplementary Tables 19–22).

### Comparative Analysis of Anti-viral Genes Expression in Melanoma Cell Lines Treated With Different DHAs

To compare the immunomodulatory activity of first and next generation DHAs, relative quantitative real-time RT-PCR analyses for HERVs expression were performed on 14 melanoma and 10 hematological cancer cell lines, respectively, and for ISGs expression on 14 melanoma cell lines, treated with 1 μM guadecitabine, DAC or AZA.

**FIGURE 4:**
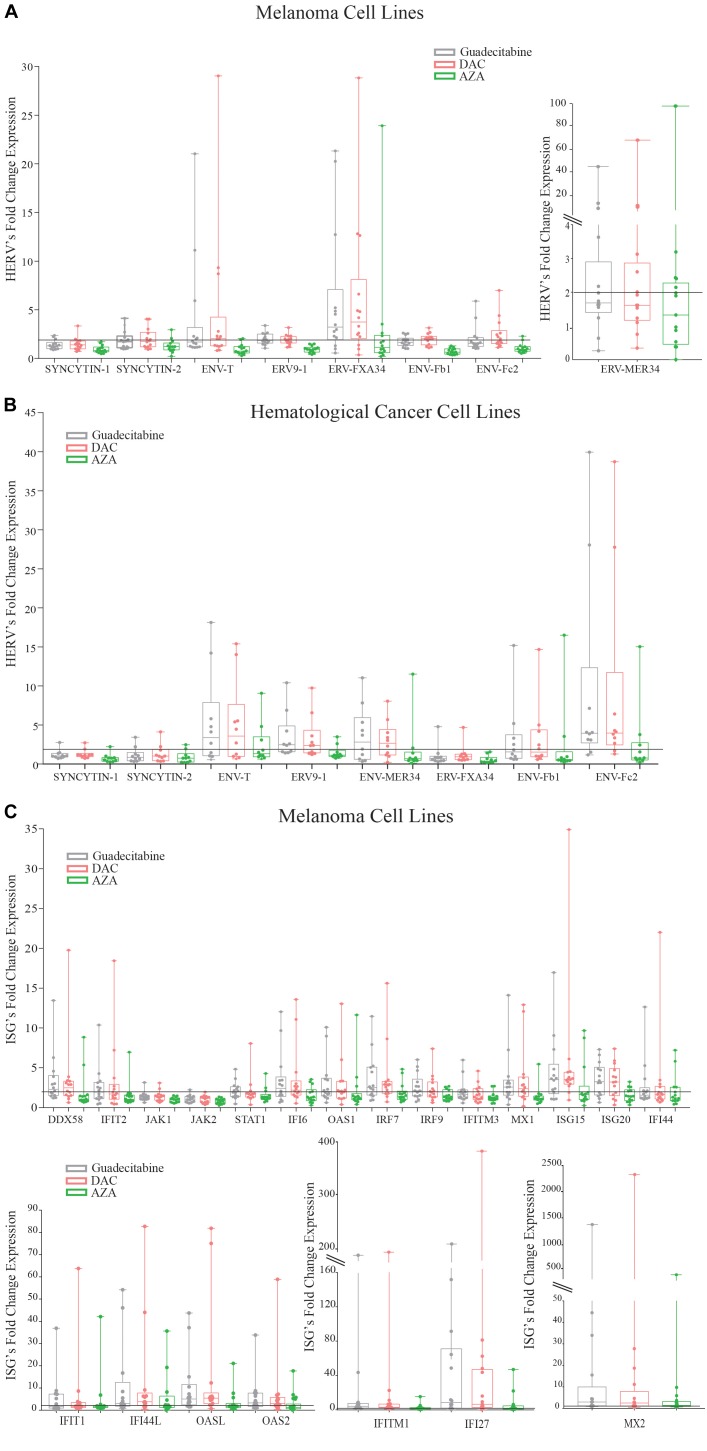
Analysis of anti-viral genes expression in melanoma cell lines treated with different DHAs. Total RNA was extracted from 14 melanoma **(A)** and 10 hematological cancer **(B)** cell lines treated with 1 μM guadecitabine (gray), DAC (pink) or AZA (green). Quantitative real-time RT-PCR analyses were performed on retrotranscribed total RNA. Box plots represent FC of HERV expression in DHAs-treated vs. untreated melanoma **(A)** and hematological cancer **(B)** cell lines and of ISG expression in DHAs-treated vs. untreated melanoma cells **(C)**. Figure shows standard deviation, median value (–), 25th Percentile, 75th percentile. Each data point represents individual cell line. Solid line (black) represents a FC expression value ≥2.

An up-regulation (FC ≥2) of the expression levels of 8 HERV genes was observed with a higher frequency in guadecitabine- or DAC- vs. AZA-treated melanoma cells: 21.4% and 7.1% vs. 0% (Syncytin-1), 28.5% and 35.7% vs. 14.2% (Syncytin-2), 35.7% and 57.1% vs. 7.1% (ENV-T), 42.8% and 42.8% vs. 0% (ERV9-1), 35.7% and 42.8% vs. 35.7% (ENV-MER34), 78.5% and 78.5% vs. 28.5% (ERV-FXA34), 21.4% and 64.2% vs. 0% (ENV-Fb1) and 28.5% and 50% vs. 7.1% (ENV-Fc2). Also in hematological cancer cell lines, HERVs up-regulation (FC ≥2) was observed with a higher frequency in guadecitabine- or DAC- vs. AZA-treated cells being 10% and 10% vs. 10% (Syncytin-1), 20% and 20% vs. 10% (Syncytin-2), 60% and 60% vs. 30% (ENV-T), 60% and 60% vs. 20% (ERV9-1), 50% and 60% vs. 20% (ENV-MER34), 10% and 10% vs. 0% (ENV-FXA34), 40% and 40% vs. 20% (ENV-Fb1) and 80% and 80% vs. 30% (ENV-Fc2). FC of investigated HERVs expression in DHAs-treated vs. untreated melanoma and hematological tumor cells are illustrated in Figures 4A,B, respectively.

Consistently, 18 out of 21 investigated ISGs were up-regulated (FC ≥2) with a higher frequency in melanoma cells treated with guadecitabine or DAC, compared to AZA. In detail, ISGs were up-regulated in 8 and 8 vs. 2 (DDX58); 7 and 6 vs. 3 (IFIT1); 7 and 5 vs. 1 (IFIT2); 1 and 2 vs. 0 (JAK1); 1 and 0 vs. 0 (JAK2); 7 and 3 vs. 3 (STAT1); 11 and 11 vs. 6 (IFI27); 8 and 8 vs. 4 (IFI6); 7 and 8 vs. 3 (OAS1); 10 and 11 vs. 5 (OAS2); 11 and 13 vs. 5 (OASL); 9 and 10 vs. 3 (IRF7); 8 and 7 vs. 4 (IRF9); 12 and 12 vs. 5 (IFITM1); 4 and 5 vs. 2 (IFITM3); 8 and 9 vs. 1 (MX1); 8 and 8 vs. 6 (MX2); 10 and 12 vs. 3 (ISG15); 9 and 9 vs. 5 (ISG20); 5 and 4 vs. 4 (IFI44) and 9 and 10 vs. 7 (IFI44L) melanoma cells, treated with guadecitabine or DAC vs. AZA, respectively. FC of investigated ISGs expression in DHAs-treated vs. untreated melanoma cells are illustrated in Figure 4C.

## Discussion

The demonstrated immunomodulatory activity of DHAs, which improves immunogenicity and immune recognition of cancer cells, results in priming and sensitizing the host immune response to immunotherapies. In light of these considerations, several clinical studies are investigating the combination of DHAs with different IC blocking mAbs in tumors of different histotype. To optimize the therapeutic efficacy of these new promising combination therapeutic strategies, we performed a comparative study of the immunomodulatory properties of selected clinically approved DHAs, AZA and DAC, and of the next generation DHA, guadecitabine, on human melanoma and hematological cancer cell lines, to identify the best epigenetic partner to be combined with immunotherapy.

The first evidence emerging from our results is a different hypomethylating effect of investigated DHAs on tumors of different histotypes. The highest LINE-1 global demethylation is achieved with guadecitabine in both melanoma and hematological cancer cell lines. A different hypomethylating effect between DHAs was already discussed in AML cells (Flotho et al., 2009; Hollenbach et al., 2010; Srivastava et al., 2014), suggesting the distinction of investigated DHAs as non-equivalent agents.

Different data support the role of epigenetic compounds in facilitating immunological targeting of cancer cells due to their ability to modulate different molecules and pathways involved in the interplay between tumor cells and the immune system (Sigalotti et al., 2014). Based on this evidence, we demonstrate the higher immunomodulatory activity of guadecitabine or DAC, compared to AZA, in reverting the CTA-negative phenotype without differences among histotypes analyzed. In particular, the higher levels of CTAs expression observed after guadecitabine or DAC treatment vs. AZA, represent an important benefit for immune recognition of cancer cells, as CTAs are able to induce both humoral and cell-mediated immune responses (Sigalotti et al., 2005), thus representing ideal targets for tumor immunotherapeutic approaches. This immunomodulatory property of guadecitabine or DAC could render tumor cells more susceptible to vaccination-stimulated CTA-specific immune responses, and more generally to CD8+ T cell specific recognition. A stronger immunomodulatory effect by guadecitabine or DAC vs. AZA treatment is observed also in the up-regulation of both HLA class I antigens, playing a central role in the presentation of TAA peptides to CTL, and of the co-stimulatory molecule, ICAM-1, allowing an increased recognition of cancer cells and promoting the activation of T cells.

Noteworthy, in addition to the above reported effects on adaptive immunity by DHAs, recent data indicated that epigenetic drugs may be exploited to allow the tumor cells eradication by innate immune system (Kima et al., 2014; Sigalotti et al., 2014). In this context, the expression of the NKG2DL MICB on melanoma cells induced only by guadecitabine and DAC treatment, could contribute to the immune recognition of transformed cells and accordingly, to their apoptosis.

Anti-tumor immunity within the TME can be supported by immune-stimulatory cytokines, such as CXCL9 and CXCL10, involved in the recruitment of immunological infiltrates at tumor site. A positive modulation of these pro-inflammatory Th1 cytokines by DHAs was previously described in ovarian cancer (Peng et al., 2015) and in epithelial cancer cell lines (Wolff et al., 2017; Lai et al., 2018); in this respect, our results underline the strongest effect of guadecitabine and DAC compared to AZA in the modulation of these cytokines, in both investigated tumor histotypes, suggesting their major contribute to the development of anti-tumor immune response.

Epigenetic activation of immune response has been recently demonstrated also through the IFN pathway signaling, upstream of antigen processing and presentation genes machinery (Chiappinelli et al., 2017). In detail, DAC and AZA primed ISGs expression in ovarian and colon cancer cells through the activation of double strand RNA derived from HERVs. Our study confirms these data in melanoma and hematological cancer cells, but also demonstrates that guadecitabine and DAC, compared to AZA, up-regulate a higher “viral mimicry” state that could eventually increase immune response. In addition, DNA demethylation offers the possibility to restore and/or to up-regulate the immunogenic potential of cancer cells, making them better targets for immunotherapeutic approaches. In this context, an important way in which DHAs may sensitize tumor cells to IC blocking therapy is through the up-regulation of immune tolerance ligands (Wrangle et al., 2013). Targeting of CTLA-4 or PD-1/PD-L1 molecules has profoundly improved the clinical management of advanced disease in a wide range of solid malignancies (Hu-Lieskovan and Ribas, 2017). In line with these evidence, we demonstrate that guadecitabine or DAC, compared to AZA, strongly up-regulate the IC mRNA expression in all investigated tumor histotypes.

The translational relevance of the immunomodulatory activities of DHAs in cancer is sustained by the results from our previous studies, in a syngeneic mouse tumor model, demonstrating how guadecitabine or DAC were able to sensitize tumor cells to the anti-tumor activity of CTLA-4 blockade, inducing a significantly stronger tumor growth reduction compared to treatment with single agents (Covre et al., 2015a,b). The immunologic aspect of the anti-tumor effects induced by DHAs in combination with IC blocking therapy was demonstrated by the highest degree of CD3 infiltrating T cells, including both CD8+ and CD4+ T cells, detected in tumors from mice treated with the combination regimen (Covre et al., 2015b).

Comprehensively, this study shows that guadecitabine has similar immunomodulatory effects to DAC and both these compounds work better compared to AZA, identifying these two drugs as optimal partners to potentiate the anti-tumor activity of different immunotherapeutic approaches, not only in solid but also in hematological tumors. The higher resistance of guadecitabine to degradation by cytidine deaminase, supports its promising clinical activity and acceptable safety profile, by prolonging its *in vivo* exposure (Roboz et al., 2018). Along this line, the ongoing NIBIT-M4 clinical study, testing the immunologic and clinical efficacy of guadecitabine combined with the anti-CTLA-4 mAb, ipilimumab, in metastatic melanoma patients (Di Giacomo et al., 2018), will provide further support to the therapeutic potential of epigenetically based immunotherapy.

## Author Contributions

CF, AC, and SCo designed and supervised the experiments. CF, OC, ML, and CC performed the experiments. PTu, SCa, and GG supported the experimental procedures. CF, AC, and SCo analyzed, interpreted the data, and wrote the manuscript. MM, ADG, PTa, JL, and RF critically revised the manuscript.

## Conflict of Interest Statement

MM is a consultant/advisory board member for Bristol-Meyers Squibb, Incyte, MSD Oncology, Roche, Astex Pharmaceuticals, Amgen, AstraZeneca, and Merck Serono. ADG has served as a consultant and/or advisor for Incyte, Pierre Fabre, and GSK. JL and RF are employed by Astex Pharmaceuticals, Inc. PTa was previously employed by Astex Pharmaceuticals, Inc. The remaining authors declare that the research was conducted in the absence of any commercial or financial relationships that could be construed as a potential conflict of interest.
