# Supplementary material for: Immunomodulatory Properties of DNA Hypomethylating Agents: Selecting the Optimal Epigenetic Partner for Cancer Immunotherapy
Source: Front Pharmacol. 2018 Dec 7;9:1443. doi: 10.3389/fphar.2018.01443 (PMC6293200; doi:10.3389/fphar.2018.01443)
Supplement: Supplementary file 1 [file Data_Sheet_1.docx]

**SUPPLEMENTARY MATERIALS**

**Supplementary Table 1. Primer sequences sets for quantitative real-time RT-PCR analysis.**

|  | **Forward sequence** | **Reverse sequence** |
| --- | --- | --- |
| β-actin | 5’-CGAGCGCGGCTACAGCTT-3’ | 5’-CCTTAATGTCACGCACGATT-3’ |
| NY-ESO-1 | 5’-TGCTTGAGTTCTACCTCGCCA-3’ | 5’-TATGTTGCCGGACACAGTGAA-3’ |
| MAGE-A3 | 5’-TGTCGTCGGAAATTGGCAGTAT-3’ | 5’-CAAAGACCAGCTGCAAGGAACT-3’ |
| MAGE-A1 | 5’-GCCAAGCACCTCTTGTATCCTG-3’ | 5’-GGAGCAGAAAACCAACCAAATC-3’ |
| CTLA-4 | 5’-TCACAGCTGTTTCTTTGAGCA-3’ | 5’-AGGCTGAAATTGCTTTTCACA-3’ |
| PD-1 | 5’-CTCAGGGTGACAGAGAGAAG-3’ | 5’-GACACCAACCACCAGGGTTT-3’ |
| PD-L1 | 5’-GGCATCCAAGATACAAACTCAA-3’ | 5’-CAGAAGTTCCAATGCTGGATTA-3’ |
| CXCL10 | 5’-CTGACTCTAAGTGGCATTCAAGGA-3’ | 5’-CAATGATCTCAACACGTGGACAA-3’ |
| CXCL9 | 5’-CCTTCCTGCGAGAAAATTGA-3’ | 5’-GCTGACCTGTTTCTCCCACT-3’ |
| MICA | 5’CCTTGGCCATGAACGTCAGG-3’ | 5’-CCTCTGAGGCCTCGCTGCG-3’ |
| MICB | 5’-AGGAGAGGAGCAGAGGTTCAC-3’ | 5’-TGGCATAGCAGCAGAAACATA-3’ |

**Supplementary Table 2. Primer sequences sets for RT-PCR analysis.**

|  | **Forward sequence** | **Reverse sequence** |
| --- | --- | --- |
| β-actin | 5’-GGCATCGTGATGGACTCCG-3’ | 5’-GCTGGAAGGTGGACAGCGA-3’ |
| MAGE-A2 | 5’-AAGTAGGACCCGAGGCACTG-3’ | 5’-GAAGAGGAAGAAGCGGTCTG-3’ |
| MAGE-A4 | 5’-GAGCAGACAGGCCAACCG-3’ | 5’-AAGGACTCTGCGTCAGGC-3’ |
| MAGE-A10 | 5’-GGAACCCCTCTTTTCTACAGAC-3’ | 5’-TCCTCTGGGGTGCTTGGTATTA-3’ |
| GAGE1-2 | 5’-GACCAAGACGCTACGTAG-3’ | 5’-CCATCAGGACCATCTTCA-3’ |
| SSX1-2 | 5’-GTGCTCAAATACCAGAGAAGATC-3’ | 5’-TTTTGGGTCCAGATCTCTCGTG-3’ |
| SSX1-5 | 5’-ACGGATCCCGTGCCATGAACGGAGACGAC-3’ | 5’-TTGTCGACAGCCATGCCCATGTTCGTGA-3’ |

**Supplementary Table 3. qRT-PCR analysis of NY-ESO-1 expression in DHAs-treated melanoma cell lines.**

| Melanoma  cell lines | CTRL | Guadecitabine | FC *vs.*  CTRL | DAC | FC *vs.*  CTRL | AZA | FC *vs.*  CTRL |
| --- | --- | --- | --- | --- | --- | --- | --- |
| Mel 195 | 1.00E-07 | **1.45E-03**^a^ | nd | **1.38E-03** | nd | 6.99E-05 | nd |
| Mel 313 | 1.00E-07 | **5.74E-04** | nd | **5.10E-04** | nd | 1.22E-05 | nd |
| Mel 275 | 1.21E-06 | **2.82E-03** | nd | **2.55E-03** | nd | **5.74E-04** | nd |
| Mel 346 | 4.99E-06 | **7.46E-04** | nd | **1.62E-03** | nd | 1.66E-05 | nd |
| Mel 116 | 2.33E-05 | **6.05E-04** | nd | **3.27E-03** | nd | 7.32E-05 | nd |
| Mel 120 | 6.96E-06 | **3.84E-04** | nd | **4.18E-04** | nd | 1.01E-05 | nd |
| Mel 514 | 9.90E-05 | **3.82E-03** | nd | **5.99E-03** | nd | **1.56E-04** | nd |
| Mel 142 | 5.89E-05 | **1.15E-01** | nd | **9.28E-02** | nd | **9.23E-03** | nd |
| Mel 237 | 1.10E-03 | 1.18E-02 | **10.71**^b^ | 8.07E-03 | **7.33** | 3.68E-03 | **3.34** |
| Mel 403 | 9.91E-03 | 5.16E-02 | **5.21** | 2.40E-02 | **2.42** | 3.65E-03 | 0.37 |
| Mel 458 | 9.15E-06 | **2.00E-03** | nd | **1.01E-03** | nd | **5.22E-04** | nd |
| Mel 345 | 9.74E-06 | **1.19E-02** | nd | **9.73E-03** | nd | **3.19E-03** | nd |
| Mel 599 | 7.71E-05 | **2.85E-04** | nd | **3.69E-04** | nd | 9.84E-05 | nd |
| Mel 261 | 4.51E-06 | 7.15E-05 | nd | 6.80E-05 | nd | 5.32E-06 | nd |
| Mean | 8.08E-04^c^ | 1.45E-02 |  | 1.08E-02 |  | 1.52E-03 |  |
| SD | 2.64E-03 | 3.19E-02 |  | 2.44E-02 |  | 2.64E-03 |  |
| Dunn Test  *vs.* CTRL |  | *p*<0.0001 |  | *p*<0.0001 |  | *p=*0.2369 |  |

^a^, In bold gene expression induced (NY-ESO-1/β-actin molecules ≥1E-04) in constitutively NY-ESO-1-negative cells; ^b^, in bold gene expression up-regulated (FC≥2) in constitutively NY-ESO-1-positive cells; ^c^, mean value of all investigated cell lines; nd, not detected.

**Supplementary Table 4. qRT-PCR analysis of MAGE-A3 expression in DHAs-treated melanoma cell lines.**

| Melanoma  cell lines | CTRL | Guadecitabine | FC *vs.*  CTRL | DAC | FC *vs.*  CTRL | AZA | FC *vs.*  CTRL |
| --- | --- | --- | --- | --- | --- | --- | --- |
| Mel 195 | 4.35E-05 | **8.40E-04**^a^ | nd | **8.91E-04** | nd | **1.35E-04** | nd |
| Mel 313 | 6.76E-05 | **4.28E-04** | nd | **4.00E-04** | nd | **1.23E-04** | nd |
| Mel 275 | 1.25E-02 | 1.47E-02 | 1.18 | 1.30E-02 | 1.04 | 1.83E-02 | 1.46 |
| Mel 346 | 7.68E-02 | 3.72E-02 | 0.48 | 1.70E-01 | **2.21** | 5.75E-02 | 0.75 |
| Mel 116 | 8.55E-02 | 9.91E-02 | 1.16 | 1.50E-01 | 1.75 | 8.16E-02 | 0.95 |
| Mel 120 | 7.71E-02 | 3.99E-02 | 0.52 | 5.62E-02 | 0.73 | 5.83E-02 | 0.76 |
| Mel 514 | 1.98E-01 | 1.34E-01 | 0.68 | 1.76E-01 | 0.89 | 1.22E-01 | 0.61 |
| Mel 142 | 3.03E-01 | 3.51E-01 | 1.16 | 3.70E-01 | 1.22 | 2.63E-01 | 0.87 |
| Mel 237 | 3.32E-01 | 2.18E-01 | 0.66 | 2.19E-01 | 0.66 | 1.97E-01 | 0.59 |
| Mel 403 | 1.55E-01 | 1.38E-01 | 0.89 | 1.40E-01 | 0.90 | 1.38E-01 | 0.89 |
| Mel 458 | 2.43E-02 | 2.68E-02 | 1.10 | 2.41E-02 | 0.99 | 2.61E-02 | 1.07 |
| Mel 345 | 2.06E-02 | 3.47E-02 | 1.69 | 4.11E-02 | 2.00 | 3.69E-02 | 1.79 |
| Mel 599 | 1.44E-03 | 3.33E-03 | **2.32**^b^ | 3.97E-03 | **2.75** | 1.88E-03 | 1.30 |
| Mel 261 | 9.76E-03 | 1.48E-02 | 1.52 | 1.37E-02 | 1.40 | 2.55E-02 | **2.61** |
| Mean | 9.26E-02^c^ | 7.95E-02 |  | 9.85E-02 |  | 7.32E-02 |  |
| SD | 1.13E-01 | 1.02E-01 |  | 1.10E-01 |  | 7.98E-02 |  |
| Dunn Test  *vs.* CTRL |  | *p*>0.9999 |  | *p=*0.2369 |  | *p>*0.9999 |  |

^a^, In bold gene expression induced (MAGE-A3/β-actin molecules ≥1E-04) in constitutively MAGE-A3-negative cells; ^b^, in bold gene expression up-regulated (FC≥2) in constitutively MAGE-A3-positive cells; ^c^, mean value of all investigated cell lines; nd, not detected.

**Supplementary Table 5. qRT-PCR analysis of MAGE-A1 expression in DHAs-treated melanoma cell lines.**

| Melanoma  cell lines | CTRL | Guadecitabine | FC *vs.*  CTRL | DAC | FC *vs.*  CTRL | AZA | FC *vs.*  CTRL |
| --- | --- | --- | --- | --- | --- | --- | --- |
| Mel 195 | 1.11E-04 | 2.54E-03 | **22.82**^b^ | 2.21E-03 | **19.86** | 6.78E-04 | **6.09** |
| Mel 313 | 1.89E-06 | **8.96E-04**^a^ | nd | **8.13E-04** | nd | 1.64E-05 | nd |
| Mel 275 | 1.51E-06 | **2.77E-03** | nd | **2.29E-03** | nd | **5.22E-04** | nd |
| Mel 346 | 8.80E-03 | 6.60E-03 | 0.75 | 1.81E-02 | **2.05** | 7.99E-03 | 0.91 |
| Mel 116 | 3.31E-03 | 6.02E-03 | 1.82 | 1.20E-02 | **3.63** | 8.87E-03 | **2.68** |
| Mel 120 | 1.93E-03 | 1.76E-03 | 0.91 | 1.71E-03 | 0.89 | 2.10E-03 | 1.09 |
| Mel 514 | 7.08E-03 | 1.18E-02 | 1.67 | 1.89E-02 | **2.66** | 7.25E-03 | 1.02 |
| Mel 142 | 3.28E-01 | 3.88E-01 | 1.18 | 4.06E-01 | 1.24 | 3.18E-01 | 0.97 |
| Mel 237 | 3.25E-01 | 3.65E-01 | 1.12 | 3.26E-01 | 1.00 | 2.61E-01 | 0.80 |
| Mel 403 | 7.10E-02 | 6.41E-02 | 0.90 | 6.43E-02 | 0.91 | 7.71E-02 | 1.09 |
| Mel 458 | 5.55E-02 | 7.50E-02 | 1.35 | 3.95E-02 | 0.71 | 7.96E-02 | 1.44 |
| Mel 345 | 1.55E-03 | 6.81E-03 | **4.38** | 4.68E-03 | **3.01** | 6.98E-03 | **4.49** |
| Mel 599 | 6.71E-05 | **1.97E-04** | nd | **2.92E-04** | nd | **1.15E-04** | nd |
| Mel 261 | 1.25E-04 | 7.04E-04 | **5.65** | 6.76E-04 | **5.42** | 5.68E-04 | **4.56** |
| Mean | 5.73E-02^c^ | 6.66E-02 |  | 6.41E-02 |  | 5.50E-02 |  |
| SD | 1.16E-01 | 1.34E-01 |  | 1.30E-01 |  | 1.03E-01 |  |
| Dunn Test  *vs.* CTRL |  | *p=*0.0252 |  | *p=*0.0252 |  | *p=*0.2369 |  |

^a^, In bold gene expression induced (MAGE-A1/β-actin molecules ≥1E-04) in constitutively MAGE-A1-negative cells; ^b^, in bold gene expression up-regulated (FC≥2) in constitutively MAGE-A1-positive cells; ^c^, mean value of all investigated cell lines; nd, not detected.

**Supplementary Table 6. qRT-PCR analysis of NY-ESO-1 expression in DHAs-treated hematological tumor cell lines.**

| Hematological tumor cell lines | CTRL | Guadecitabine | DAC | AZA |
| --- | --- | --- | --- | --- |
| Daudi | 3.64E-06 | **2.84E-02**^a^ | **2.60E-02** | **3.73E-03** |
| HL-60 | 1.59E-06 | **1.50E-03** | **6.45E-04** | 5.89E-05 |
| NALM-6 | 1.07E-06 | **5.80E-03** | **2.79E-03** | **3.02E-03** |
| Rajy | 3.05E-06 | **4.79E-03** | **3.21E-03** | **3.44E-03** |
| U-937 | 8.60E-07 | **7.00E-03** | **6.81E-03** | **1.80E-03** |
| KG-1a | 7.57E-07 | **8.41E-03** | **1.24E-02** | **1.38E-03** |
| JURKAT | 2.87E-05 | **2.34E-03** | **2.94E-03** | **6.73E-04** |
| K562 | 1.41E-07 | **9.42E-03** | **7.43E-03** | 9.69E-05 |
| Ri-1 | 2.02E-07 | **8.01E-03** | **5.15E-03** | **3.19E-03** |
| JY | 3.05E-07 | **4.69E-03** | **3.61E-03** | **5.19E-03** |
| Mean | 4.00E-06^b^ | 8.00E-03 | 7.10E-03 | 2.30E-03 |
| SD | 8.75E-06 | 7.61E-03 | 7.42E-03 | 1.72E-03 |
| Dunn Test *vs.* CTRL |  | *p*<0.0001 | *p=*0.003 | *p*=0.0459 |

^a^, In bold gene expression induced (NY-ESO-1/β-actin molecules ≥1E-04) in constitutively NY-ESO-1 negative cells; ^b^, mean value of all investigated cell lines.

**Supplementary Table 7. qRT-PCR analysis of MAGE-A3 expression in DHAs-treated hematological tumor cell lines.**

| Hematological  tumor cell lines | CTRL | Guadecitabine | FC *vs.*  CTRL | DAC | FC *vs.*  CTRL | AZA | FC *vs.*  CTRL |
| --- | --- | --- | --- | --- | --- | --- | --- |
| Daudi | 2.13E-05 | **4.64E-02**^a^ | nd | **4.12E-02** | nd | **1.60E-02** | nd |
| HL-60 | 5.78E-06 | **9.33E-04** | nd | **6.97E-04** | nd | **2.69E-04** | nd |
| NALM-6 | 3.78E-06 | **2.76E-02** | nd | **2.53E-02** | nd | **1.52E-02** | nd |
| Rajy | 3.93E-06 | **3.03E-02** | nd | **1.97E-02** | nd | **1.42E-02** | nd |
| U-937 | 1.73E-06 | **2.15E-04** | nd | **3.13E-04** | nd | 5.80E-05 | nd |
| KG-1a | 2.07E-06 | **3.52E-04** | nd | **6.50E-04** | nd | 5.63E-05 | nd |
| JURKAT | 1.31E-06 | **2.28E-02** | nd | **2.13E-02** | nd | **1.17E-02** | nd |
| K562 | 2.32E-02 | 1.20E-01 | **5.15**^b^ | 9.72E-02 | **4.18** | 1.29E-01 | **5.57** |
| Ri-1 | 5.69E-06 | **3.08E-02** | nd | **2.28E-02** | nd | **1.56E-02** | nd |
| JY | 5.87E-05 | **1.23E-02** | nd | **9.53E-03** | nd | **8.53E-03** | nd |
| Mean | 2.30E-03^c^ | 2.90E-02 |  | 2.40E-02 |  | 2.10E-02 |  |
| SD | 7.35E-03 | 3.54E-02 |  | 2.89E-02 |  | 3.86E-02 |  |
| Dunn Test  *vs.* CTRL |  | *p*<0.0001 |  | *p=*0.0008 |  | *p=*0.113 |  |

^a^, In bold gene expression induced (MAGE-A3/β-actin molecules ≥1E-04) in constitutively MAGE-A3-negative cells; ^b^, in bold gene expression up-regulated (FC≥2) in constitutively MAGE-A3-positive cells; ^c^, mean value of all investigated cell lines; nd, not detected.

**Supplementary Table 8. qRT-PCR analysis of MAGE-A1 expression in DHAs-treated hematological tumor cell lines.**

| Hematological  tumor cell lines | CTRL | Guadecitabine | FC *vs.*  CTRL | DAC | FC *vs.*  CTRL | AZA | FC *vs.*  CTRL |
| --- | --- | --- | --- | --- | --- | --- | --- |
| Daudi | 2.07E-05 | **1.39E-02**^a^ | nd | **1.45E-02** | nd | **9.27E-03** | nd |
| HL-60 | 2.00E-05 | **1.35E-03** | nd | **7.10E-04** | nd | **2.64E-04** | nd |
| NALM-6 | 2.90E-05 | **1.27E-02** | nd | **9.73E-03** | nd | **1.03E-02** | nd |
| Rajy | 2.50E-05 | **1.38E-02** | nd | **8.86E-03** | nd | **9.49E-03** | nd |
| U-937 | 1.55E-06 | 7.54E-06 | nd | 8.89E-06 | nd | 4.42E-06 | nd |
| KG-1a | 2.98E-06 | 7.81E-05 | nd | **2.20E-04** | nd | 2.22E-05 | nd |
| JURKAT | 4.71E-05 | **4.20E-02** | nd | **3.71E-02** | nd | **2.75E-02** | nd |
| K562 | 6.84E-03 | 3.76E-02 | **5.49**^b^ | 2.82E-02 | **4.12** | 3.48E-02 | **5.08** |
| Ri-1 | 1.37E-05 | **1.55E-02** | nd | **1.05E-02** | nd | **1.11E-02** | nd |
| JY | 5.28E-06 | **1.25E-03** | nd | **9.18E-04** | nd | **1.01E-03** | nd |
| Mean | 7.01E-04^c^ | 1.38E-02 |  | 1.11E-02 |  | 1.04E-02 |  |
| SD | 2.16E-03 | 1.51E-02 |  | 1.26E-02 |  | 1.20E-02 |  |
| Dunn Test  *vs.* CTRL |  | *p*<0.0001 |  | *p=*0.0055 |  | *p=*0.0281 |  |

^a^, In bold gene expression induced (MAGE-A1/β-actin molecules ≥1E-04) in constitutively MAGE-A1-negative cells; ^b^, in bold gene expression up-regulated (FC≥2) in constitutively MAGE-A1-positive cells; ^c^, mean value of all investigated cell lines; nd, not detected.

**Supplementary Table 9. qRT-PCR analysis of CTLA-4 expression in DHAs-treated melanoma cell lines.**

| Melanoma  cell lines | CTRL | Guadecitabine | FC *vs.*  CTRL | DAC | FC *vs.*  CTRL | AZA | FC *vs.*  CTRL |
| --- | --- | --- | --- | --- | --- | --- | --- |
| Mel 195 | 1.35E-02 | 6.14E-03 | 0.46 | 1.26E-02 | 0.94 | 1.36E-03 | 0.10 |
| Mel 313 | 6.45E-06 | 2.52E-05 | nd | **3.22E-05** | nd | 4.63E-06 | nd |
| Mel 275 | 8.87E-04 | 7.08E-04 | 0.80 | 6.45E-03 | **7.27** | 2.27E-03 | **2.54** |
| Mel 346 | 1.15E-06 | 3.14E-05 | nd | 2.38E-04 | nd | 1.77E-06 | nd |
| Mel 116 | 4.19E-04 | 1.93E-03 | **4.60**^b^ | 3.12E-03 | **7.27** | 1.82E-03 | **2.56** |
| Mel 120 | 1.27E-05 | **1.01E-04**^a^ | nd | **1.91E-04** | nd | 4.91E-05 | nd |
| Mel 514 | 3.31E-05 | **7.12E-04** | nd | **1.06E-03** | nd | 2.67E-05 | nd |
| Mel 142 | 1.24E-01 | 1.59E-01 | 1.28 | 1.90E-01 | 1.53 | 8.78E-02 | 0.71 |
| Mel 237 | 6.26E-02 | 1.55E-01 | **2.48** | 1.80E-01 | **2.88** | 7.20E-02 | 1.15 |
| Mel 403 | 3.06E-02 | 2.40E-01 | **7.86** | 1.16E-01 | **3.79** | 3.79E-03 | 0.12 |
| Mel 458 | 6.06E-05 | **4.08E-04** | nd | **2.52E-04** | nd | **1.96E-04** | nd |
| Mel 345 | 2.59E-05 | **4.22E-03** | nd | **6.47E-03** | nd | **3.50E-04** | nd |
| Mel 599 | 5.19E-03 | 1.00E-02 | 1.93 | 1.16E-02 | **2.23** | 7.87E-03 | 1.52 |
| Mel 261 | 4.25E-06 | **3.53E-04** | nd | **4.06E-04** | nd | **1.45E-04** | nd |
| Mean | 1.70E-02^c^ | 4.14E-02 |  | 3.78E-02 |  | 1.27E-02 |  |
| SD | 3.55E-02 | 8.01E-02 |  | 6.94E-02 |  | 2.87E-02 |  |
| Dunn Test  *vs.* CTRL |  | *p=*0.0162 |  | *p*<0.0001 |  | *p*>0.9999 |  |

^a^, In bold gene expression induced (CTLA-4/β-actin molecules ≥1E-04) in constitutively CTLA-4-negative cells; ^b^, in bold gene expression up-regulated (FC≥2) in constitutively CTLA-4-positive cells; ^c^, mean value of all investigated cell lines; nd, not detected.

**Supplementary Table 10. qRT-PCR analysis of PD-1 expression in DHAs-treated melanoma cell lines.**

| Melanoma cell lines | CTRL | Guadecitabine | DAC | AZA |
| --- | --- | --- | --- | --- |
| Mel 195 | 8.87E-07 | 4.58E-05 | 7.15E-05 | 7.91E-06 |
| Mel 313 | 4.13E-06 | **3.39E-04**^a^ | **3.71E-04** | 6.35E-06 |
| Mel 275 | 1.63E-06 | **2.45E-04** | **2.74E-04** | 4.35E-05 |
| Mel 346 | 5.09E-07 | 1.42E-05 | 1.08E-05 | 1.54E-07 |
| Mel 116 | 1.13E-06 | 9.73E-06 | 2.97E-05 | 3.15E-06 |
| Mel 120 | 1.02E-07 | 1.03E-06 | 1.13E-06 | 8.44E-08 |
| Mel 514 | 4.18E-07 | 1.12E-05 | 1.86E-05 | 1.29E-06 |
| Mel 142 | 4.11E-07 | **1.58E-04** | **2.21E-04** | 8.11E-06 |
| Mel 237 | 6.42E-05 | **1.36E-03** | **1.69E-03** | **1.87E-04** |
| Mel 403 | 5.11E-06 | 1.09E-05 | 1.29E-05 | 3.29E-07 |
| Mel 458 | 2.89E-06 | 5.77E-05 | **1.17E-04** | 4.75E-06 |
| Mel 345 | 2.81E-07 | 3.49E-05 | **1.48E-04** | 5.38E-06 |
| Mel 599 | 2.13E-07 | 3.03E-05 | 3.55E-05 | 7.81E-07 |
| Mel 261 | 3.10E-07 | 9.71E-06 | 7.91E-06 | 1.13E-06 |
| Mean | 5.87E-06^b^ | 1.66E-04 | 2.15E-04 | 1.92E-05 |
| SD | 1.69E-05 | 3.59E-04 | 4.39E-04 | 4.94E-05 |
| Dunn Test *vs.* CTRL |  | *p=*0.0002 | *p*<0.0001 | *p=*0.7247 |

^a^, In bold gene expression induced (PD-1/β-actin molecules ≥1E-04) in constitutively PD-1-negative cells; ^b^, mean value of all investigated cell lines.

**Supplementary Table 11. qRT-PCR analysis of PD-L1 expression in DHAs-treated melanoma cell lines.**

| Melanoma  cell lines | CTRL | Guadecitabine | FC *vs.*  CTRL | DAC | FC *vs.*  CTRL | AZA | FC *vs.*  CTRL |
| --- | --- | --- | --- | --- | --- | --- | --- |
| Mel 195 | 4.04E-04 | 1.65E-03 | **4.10**^a^ | 2.02E-03 | **5.01** | 1.91E-03 | **4.74** |
| Mel 313 | 1.79E-03 | 1.94E-03 | 1.08 | 1.72E-03 | 0.96 | 1.21E-03 | 0.67 |
| Mel 275 | 2.41E-04 | 3.54E-04 | 1.47 | 3.58E-04 | 1.48 | 1.41E-04 | 0.58 |
| Mel 346 | 1.07E-04 | 2.63E-04 | **2.46** | 5.22E-04 | **4.89** | 1.32E-04 | 1.23 |
| Mel 116 | 2.60E-04 | 1.82E-04 | 0.70 | 9.30E-04 | **3.57** | 7.20E-05 | 0.28 |
| Mel 120 | 1.78E-04 | 1.60E-04 | 0.90 | 2.06E-04 | 1.16 | 1.89E-04 | 1.06 |
| Mel 514 | 2.16E-03 | 7.44E-04 | 0.34 | 1.14E-03 | 0.53 | 4.67E-04 | 0.22 |
| Mel 142 | 1.67E-04 | 5.11E-04 | **3.06** | 6.68E-04 | **4.00** | 4.64E-04 | **2.78** |
| Mel 237 | 1.77E-03 | 1.60E-03 | 0.91 | 1.81E-03 | 1.03 | 9.58E-04 | 0.54 |
| Mel 403 | 1.07E-04 | 2.20E-04 | **2.05** | 1.99E-04 | 1.85 | 4.07E-05 | 0.38 |
| Mel 458 | 1.94E-03 | 8.58E-04 | 0.44 | 6.95E-04 | 0.36 | 1.03E-03 | 0.53 |
| Mel 345 | 4.56E-04 | 1.37E-03 | **3.01** | 1.59E-03 | **3.50** | 4.66E-04 | 1.02 |
| Mel 599 | 7.45E-04 | 1.70E-03 | **2.28** | 2.21E-03 | **2.97** | 8.06E-04 | 1.08 |
| Mel 261 | 1.20E-04 | 7.33E-04 | **6.10** | 8.02E-04 | **6.68** | 5.40E-04 | **4.50** |
| Mean | 7.46E-04^b^ | 8.78E-04 |  | 1.06E-03 |  | 6.02E-04 |  |
| SD | 7.90E-04 | 6.47E-04 |  | 6.89E-04 |  | 5.34E-04 |  |
| Dunn Test  *vs.* CTRL |  | *p=*0.7247 |  | *p=*0.0102 |  | *p*>0.9999 |  |

^a^, In bold gene expression up-regulated (FC≥2) in constitutively PD-L1-positive cells; ^b^, mean value of all investigated cell lines.

**Supplementary Table 12. qRT-PCR analysis of CTLA-4 expression in DHAs-treated hematological tumor cell lines.**

| Hematological tumor cell lines | CTRL | Guadecitabine | DAC | AZA |
| --- | --- | --- | --- | --- |
| Daudi | 3.39E-06 | **2.03E-04**^a^ | **1.02E-04** | 3.83E-05 |
| HL-60 | 3.61E-06 | 6.17E-05 | 9.85E-05 | 1.99E-06 |
| NALM-6 | 6.73E-07 | 2.08E-05 | 1.68E-05 | 4.68E-06 |
| Rajy | 1.13E-06 | 2.60E-05 | 1.30E-05 | 1.83E-06 |
| U-937 | 3.03E-06 | 5.25E-05 | **1.03E-04** | 2.66E-06 |
| KG-1a | 1.30E-06 | 7.09E-05 | **1.62E-04** | 1.63E-06 |
| JURKAT | 2.65E-05 | **2.82E-03** | **3.10E-03** | **3.59E-04** |
| K562 | 1.07E-06 | 1.52E-06 | 6.89E-07 | 1.20E-06 |
| Ri-1 | 7.75E-08 | 3.70E-06 | 2.01E-06 | 5.31E-07 |
| JY | 2.27E-06 | **1.56E-04** | **1.63E-04** | **1.49E-04** |
| Mean | 4.31E-06^b^ | 3.42E-04 | 3.76E-04 | 5.61E-05 |
| SD | 7.90E-06 | 8.75E-04 | 9.58E-04 | 1.16E-04 |
| Dunn Test *vs.* CTRL |  | *p=*0.0004 | *p=*0.0016 | *p=*0.8961 |

^a^, In bold gene expression induced (CTLA-4/β-actin molecules ≥1E-04) in constitutively CTLA-4-negative cells; ^b^, mean value of all investigated cell lines.

**Supplementary Table 13. qRT-PCR analysis of PD-1 expression in DHAs-treated hematological tumor cell lines.**

| Hematological tumor cell lines | CTRL | Guadecitabine | DAC | AZA |
| --- | --- | --- | --- | --- |
| Daudi | 3.57E-06 | **4.64E-04**^a^ | **3.47E-04** | **1.23E-04** |
| HL-60 | 5.78E-07 | **1.71E-04** | **2.99E-04** | 7.13E-07 |
| NALM-6 | 3.82E-06 | 7.60E-05 | 8.15E-05 | 1.49E-05 |
| Rajy | 7.30E-07 | **1.23E-04** | 7.43E-05 | 2.40E-05 |
| U-937 | 1.24E-06 | 3.76E-05 | 4.01E-05 | 1.70E-07 |
| KG-1a | 4.58E-07 | 6.74E-05 | **1.48E-04** | 4.11E-07 |
| JURKAT | 2.38E-03 | 6.03E-03 | 7.05E-03 | 1.98E-03 |
| K562 | 1.55E-05 | 9.06E-06 | 1.72E-05 | 1.60E-05 |
| Ri-1 | 5.67E-07 | 3.36E-05 | 3.75E-05 | 1.43E-05 |
| JY | 4.08E-06 | **1.69E-04** | **1.63E-04** | **2.01E-04** |
| Mean | 2.41E-04^b^ | 7.18E-04 | 8.26E-04 | 2.38E-04 |
| SD | 7.52E-04 | 1.87E-03 | 2.19E-03 | 6.16E-04 |
| Dunn Test *vs.* CTRL |  | *p=*0.0168 | *p=*0.0004 | *p=*0.8961 |

^a^, In bold gene expression induced (PD-1/β-actin molecules ≥1E-04) in constitutively PD-1-negative cells; ^b^, mean value of all investigated cell lines.

**Supplementary Table 14. qRT-PCR analysis of PD-L1 expression in DHAs-treated hematological tumor cell lines.**

| Hematological  tumor cell lines | CTRL | Guadecitabine | FC *vs.*  CTRL | DAC | FC *vs.*  CTRL | AZA | FC *vs.*  CTRL |
| --- | --- | --- | --- | --- | --- | --- | --- |
| Daudi | 1.86E-03 | 1.43E-03 | 0.77 | 1.78E-03 | 0.96 | 1.33E-03 | 0.71 |
| HL-60 | 8.36E-06 | 4.98E-05 | nd | **1.12E-04** ^a^ | nd | 1.08E-05 | nd |
| NALM-6 | 9.58E-04 | 6.02E-03 | **6.29**^b^ | 4.83E-03 | **5.05** | 1.48E-03 | 1.54 |
| Rajy | 2.28E-03 | 5.66E-03 | **2.48** | 5.01E-03 | **2.19** | 2.11E-03 | 0.93 |
| U-937 | 1.20E-04 | 5.61E-04 | **4.68** | 5.23E-04 | **4.36** | 6.34E-05 | 0.53 |
| KG-1a | 1.13E-04 | 8.08E-04 | **7.17** | 8.43E-04 | **7.48** | 4.64E-05 | 0.41 |
| JURKAT | 6.52E-04 | 7.83E-04 | 1.20 | 1.10E-03 | 1.68 | 3.14E-04 | 0.48 |
| K562 | 4.28E-05 | 6.20E-05 | nd | 7.48E-05 | nd | 5.53E-05 | nd |
| Ri-1 | 1.63E-03 | 4.36E-03 | **2.67** | 4.30E-03 | **2.63** | 1.97E-03 | 1.21 |
| JY | 2.27E-02 | 2.21E-02 | 0.97 | 2.37E-02 | 1.04 | 2.38E-02 | 1.05 |
| Mean | 3.04E-03^c^ | 4.19E-03 |  | 4.23E-03 |  | 3.11E-03 |  |
| SD | 6.97E-03 | 6.72E-03 |  | 7.11E-03 |  | 7.30E-03 |  |
| Dunn Test  *vs.* CTRL |  | *p=*0.073 |  | *p=*0.0168 |  | *p*>0.9999 |  |

^a^, In bold gene expression induced (PD-L1/β-actin molecules ≥1E-04) in constitutively PD-L1-negative cells; ^b^, in bold gene expression up-regulated (FC≥2) in constitutively PD-L1-positive cells; ^c^, mean value of all investigated cell lines; nd, not detected.

**Supplementary Table 15. qRT-PCR analysis of CXCL10 expression in DHAs-treated melanoma cell lines.**

| Melanoma cell lines | CTRL | Guadecitabine | DAC | AZA |
| --- | --- | --- | --- | --- |
| Mel 195 | 1.11E-05 | **1.30E-03**^a^ | **2.24E-03** | **1.14E-03** |
| Mel 313 | 0.00E+00 | 2.38E-05 | 5.64E-05 | **2.05E-03** |
| Mel 275 | 1.68E-04 | 1.00E-04 | 1.18E-04 | 3.93E-05 |
| Mel 346 | 1.01E-05 | 1.94E-05 | 4.11E-05 | 1.13E-06 |
| Mel 116 | 8.64E-05 | **2.39E-04** | **5.01E-04** | 6.87E-05 |
| Mel 120 | 0.00E+00 | 3.30E-06 | 1.80E-05 | 1.70E-06 |
| Mel 514 | 1.84E-06 | 2.30E-05 | 7.36E-06 | 5.08E-06 |
| Mel 142 | 2.20E-05 | **7.63E-03** | **7.19E-03** | 3.29E-05 |
| Mel 237 | 9.63E-06 | **6.17E-04** | **2.00E-03** | 8.95E-05 |
| Mel 403 | 0.00E+00 | 6.39E-06 | 5.36E-06 | 0.00E+00 |
| Mel 458 | 0.00E+00 | 4.78E-06 | 1.22E-05 | 6.78E-07 |
| Mel 345 | 4.57E-07 | 9.28E-06 | 1.43E-05 | 1.68E-08 |
| Mel 599 | 3.79E-06 | 3.30E-05 | 4.04E-05 | 8.56E-06 |
| Mel 261 | 3.29E-06 | **1.57E-04** | 9.17E-05 | 9.95E-05 |
| Mean | 2.26E-05^b^ | 7.26E-04 | 8.81E-04 | 2.53E-04 |
| SD | 4.75E-05 | 2.02E-03 | 1.96E-03 | 5.98E-04 |
| Dunn Test *vs.* CTRL |  | *p=*0.0017 | *p*<0.0001 | *p*>0.9999 |

^a^, In bold gene expression induced (CXCL10/β-actin molecules ≥1E-04) in constitutively CXCL10-negative cells; ^b^, mean value of all investigated cell lines.

**Supplementary Table 16. qRT-PCR analysis of CXCL9 expression in DHAs-treated melanoma cell lines.**

| Melanoma  cell lines | CTRL | Guadecitabine | FC *vs.*  CTRL | DAC | FC *vs.*  CTRL | AZA | FC *vs.*  CTRL |
| --- | --- | --- | --- | --- | --- | --- | --- |
| Mel 195 | 8.19E-05 | 5.95E-05 | nd | **2.58E-04**^a^ | nd | 5.20E-05 | nd |
| Mel 313 | 9.71E-07 | 6.88E-06 | nd | 9.57E-06 | nd | 1.54E-05 | nd |
| Mel 275 | 1.68E-05 | 4.78E-05 | nd | 4.81E-05 | nd | 2.93E-05 | nd |
| Mel 346 | 1.08E-06 | 1.82E-06 | nd | 1.22E-05 | nd | 5.27E-07 | nd |
| Mel 116 | 3.67E-04 | 7.30E-04 | 1.99 | 8.86E-04 | **2.41** | 1.00E-04 | 0.27 |
| Mel 120 | 1.04E-04 | 3.16E-04 | **3.03**^b^ | 4.03E-04 | **3.87** | 1.30E-04 | 1.25 |
| Mel 514 | 1.26E-06 | 1.29E-05 | nd | 2.35E-05 | nd | 1.54E-06 | nd |
| Mel 142 | 4.38E-04 | 4.22E-04 | 0.96 | 4.11E-04 | 0.94 | 1.05E-04 | 0.24 |
| Mel 237 | 1.38E-04 | 2.62E-04 | 1.89 | 5.79E-04 | **4.19** | 4.29E-05 | 0.31 |
| Mel 403 | 2.15E-05 | 3.57E-05 | nd | **1.11E-04** | nd | 8.02E-06 | nd |
| Mel 458 | 2.27E-05 | 8.62E-05 | nd | 8.12E-05 | nd | 2.07E-05 | nd |
| Mel 345 | 4.83E-06 | 1.28E-05 | nd | 3.25E-05 | nd | 6.88E-06 | nd |
| Mel 599 | 3.17E-06 | 1.83E-05 | nd | 1.19E-05 | nd | 7.95E-06 | nd |
| Mel 261 | 6.21E-06 | 2.13E-05 | nd | 3.30E-05 | nd | 2.85E-06 | nd |
| Mean | 8.62E-05^c^ | 1.45E-04 |  | 2.07E-04 |  | 3.73E-05 |  |
| SD | 1.41E-04 | 2.15E-04 |  | 2.69E-04 |  | 4.35E-05 |  |
| Dunn Test  *vs.* CTRL |  | *p=*0.0385 |  | *p=*0.0004 |  | *p*>0.9999 |  |

^a^, In bold gene expression induced (CXCL9/β-actin molecules ≥1E-04) in constitutively CXCL9-negative cells; ^b^, in bold gene expression up-regulated (FC≥2) in constitutively CXCL9-positive cells; ^c^, mean value of all investigated cell lines; nd, not detected.

**Supplementary Table 17. qRT-PCR analysis of MICB expression in DHAs-treated melanoma cell lines.**

| Melanoma  cell lines | CTRL | Guadecitabine | FC *vs.*  CTRL | DAC | FC *vs.*  CTRL | AZA | FC *vs.*  CTRL |
| --- | --- | --- | --- | --- | --- | --- | --- |
| Mel 195 | 8.21E-06 | **1.98E-04**^a^ | nd | **2.01E-04** | nd | **7.13E-04** | nd |
| Mel 313 | 8.83E-04 | 1.84E-03 | **2.09**^b^ | 2.04E-03 | **2.31** | 5.67E-04 | 0.64 |
| Mel 275 | 5.23E-04 | 1.31E-03 | **2.52** | 1.32E-03 | **2.53** | 6.97E-04 | 1.33 |
| Mel 346 | 3.39E-04 | 1.01E-03 | **2.98** | 1.20E-03 | **3.55** | 4.76E-04 | 1.41 |
| Mel 116 | 2.02E-03 | 2.81E-03 | 1.39 | 4.57E-03 | **2.26** | 1.33E-03 | 0.66 |
| Mel 120 | 1.63E-03 | 1.97E-03 | 1.21 | 2.03E-03 | 1.24 | 1.85E-03 | 1.14 |
| Mel 514 | 2.14E-03 | 4.35E-03 | **2.04** | 3.39E-03 | 1.59 | 2.68E-03 | 1.25 |
| Mel 142 | 1.16E-03 | 2.95E-03 | **2.55** | 3.35E-03 | **2.89** | 1.67E-03 | 1.44 |
| Mel 237 | 4.70E-03 | 5.46E-03 | 1.16 | 7.08E-03 | 1.51 | 3.06E-03 | 0.65 |
| Mel 403 | 1.63E-03 | 2.48E-03 | 1.52 | 2.24E-03 | 1.38 | 2.10E-03 | 1.29 |
| Mel 458 | 2.48E-03 | 2.23E-03 | 0.90 | 2.08E-03 | 0.84 | 2.70E-03 | 1.09 |
| Mel 345 | 5.20E-04 | 1.30E-03 | **2.49** | 1.78E-03 | **3.41** | 1.24E-03 | **2.39** |
| Mel 599 | 5.32E-04 | 1.04E-03 | 1.95 | 1.04E-03 | 1.95 | 8.94E-04 | 1.68 |
| Mel 261 | 8.15E-04 | 2.22E-03 | **2.72** | 1.80E-03 | **2.21** | 1.39E-03 | 1.70 |
| Mean | 1.38E-03^c^ | 2.23E-03 |  | 2.44E-03 |  | 1.53E-03 |  |
| SD | 1.21E-03 | 1.38E-03 |  | 1.73E-03 |  | 8.52E-04 |  |
| Dunn Test  *vs.* CTRL |  | *p*=0.001 |  | *p*<0.0001 |  | *p*=0.4297 |  |

^a^, In bold gene expression induced (MICB/β-actin molecules ≥1E-04) in constitutively MICB-negative cells; ^b^, in bold gene expression up-regulated (FC≥2) in constitutively MICB-positive cells; ^c^, mean value of all investigated cell lines; nd, not detected.

**Supplementary Table 18. qRT-PCR analysis of MICA expression in DHAs-treated melanoma cell lines.**

| Melanoma  cell lines | CTRL | Guadecitabine | FC *vs.*  CTRL | DAC | FC *vs.*  CTRL | AZA | FC *vs.*  CTRL |
| --- | --- | --- | --- | --- | --- | --- | --- |
| Mel 195 | 1.94E-03 | 5.96E-03 | **3.08**^a^ | 6.34E-03 | **3.28** | 5.82E-03 | **3.01** |
| Mel 313 | 7.76E-03 | 6.50E-03 | 0.84 | 8.39E-03 | 1.08 | 6.84E-03 | 0.88 |
| Mel 275 | 4.61E-03 | 6.92E-03 | 1.50 | 7.94E-03 | 1.72 | 4.69E-03 | 1.02 |
| Mel 346 | 4.69E-03 | 7.72E-03 | 1.64 | 7.36E-03 | 1.57 | 6.96E-03 | 1.48 |
| Mel 116 | 1.94E-02 | 2.50E-02 | 1.29 | 3.07E-02 | 1.58 | 1.18E-02 | 0.61 |
| Mel 120 | 5.47E-04 | 5.89E-04 | 1.08 | 5.28E-04 | 0.96 | 4.23E-04 | 0.77 |
| Mel 514 | 1.40E-03 | 1.74E-03 | 1.24 | 1.78E-03 | 1.27 | 1.65E-03 | 1.18 |
| Mel 142 | 6.58E-04 | 1.07E-03 | 1.62 | 1.18E-03 | 1.79 | 8.37E-04 | 1.27 |
| Mel 237 | 7.48E-03 | 9.11E-03 | 1.22 | 1.14E-02 | 1.52 | 5.76E-03 | 0.77 |
| Mel 403 | 3.42E-03 | 4.87E-03 | 1.42 | 4.80E-03 | 1.40 | 4.79E-03 | 1.40 |
| Mel 458 | 6.12E-03 | 7.95E-03 | 1.30 | 8.18E-03 | 1.34 | 7.08E-03 | 1.16 |
| Mel 345 | 1.06E-03 | 1.38E-03 | 1.30 | 2.18E-03 | **2.06** | 1.95E-03 | 1.84 |
| Mel 599 | 1.00E-03 | 2.52E-03 | **2.51** | 1.80E-03 | 1.79 | 1.64E-03 | 1.64 |
| Mel 261 | 1.13E-03 | 1.68E-03 | 1.49 | 1.95E-03 | 1.73 | 2.24E-03 | 1.99 |
| Mean | 4.37E-03^b^ | 5.93E-03 |  | 6.75E-03 |  | 4.47E-03 |  |
| SD | 5.02E-03 | 6.23E-03 |  | 7.68E-03 |  | 3.20E-03 |  |
| Dunn Test  *vs.* CTRL |  | *p=*0.0038 |  | *p*<0.0001 |  | *p=*0.7247 |  |

^a^, In bold gene expression up-regulated (FC≥2) in constitutively MICA-positive cells; ^b^, mean value of all investigated cell lines.

**Supplementary Table 19. qRT-PCR analysis of CXCL10 expression in DHAs-treated hematological cancer cell lines.**

| Hematological  tumor cell lines | CTRL | Guadecitabine | FC *vs.*  CTRL | DAC | FC *vs.*  CTRL | AZA | FC *vs.*  CTRL |
| --- | --- | --- | --- | --- | --- | --- | --- |
| Daudi | 1.12E-03 | 2.14E-03 | 1.9 | 6.34E-03 | **5.65** | 1.82E-03 | 1.62 |
| HL-60 | 0.00E+00 | 6.25E-06 | nd | 2.41E-05 | nd | 3.68E-07 | nd |
| NALM-6 | 2.04E-04 | 1.01E-01 | **494.74**^b^ | 9.56E-02 | **468.46** | 3.62E-02 | **177.53** |
| Rajy | 6.01E-04 | 9.86E-02 | **164.03** | 8.26E-02 | **137.35** | 2.79E-02 | **46.49** |
| U-937 | 2.94E-05 | **3.67E-03**^a^ | nd | **6.45E-03** | nd | 9.44E-05 | nd |
| KG-1a | 4.77E-05 | **1.08E-02** | nd | **6.35E-03** | nd | 8.38E-05 | nd |
| JURKAT | 3.29E-07 | 5.98E-05 | nd | **1.89E-04** | nd | 6.88E-07 | nd |
| K562 | 0.00E+00 | 9.48E-07 | nd | 6.44E-08 | nd | 1.11E-06 | nd |
| Ri-1 | 1.84E-04 | 6.23E-02 | **338.70** | 6.61E-02 | **359.61** | 3.30E-02 | **179.27** |
| JY | 7.50E-05 | **5.79E-02** | nd | **8.80E-02** | nd | **4.50E-03** | nd |
| Mean | 2.26E-04^c^ | 3.37E-02 |  | 3.52E-02 |  | 1.04E-02 |  |
| SD | 3.64E-04 | 4.21E-02 |  | 4.19E-02 |  | 1.54E-02 |  |
| Dunn Test  *vs.* CTRL |  | *p*=0.4976 |  | *p>*0.9999 |  | *p*=0.0004 |  |

^a^, In bold gene expression induced (CXCL10/β-actin molecules ≥1E-04) in constitutively CXCL10-negative cells; ^b^, in bold gene expression up-regulated (FC≥2) in constitutively CXCL10-positive cells; ^c^, mean value of all investigated cell lines; nd, not detected.

**Supplementary Table 20. qRT-PCR analysis of CXCL9 expression in DHAs-treated hematological cancer cell lines.**

| Hematological  tumor cell lines | CTRL | Guadecitabine | FC *vs.*  CTRL | DAC | FC *vs.*  CTRL | AZA | FC *vs.*CTRL |
| --- | --- | --- | --- | --- | --- | --- | --- |
| Daudi | 1.03E-06 | **1.62E-04**^a^ | nd | **2.13E-04** | nd | 1.33E-05 | nd |
| HL-60 | 6.77E-07 | 4.07E-06 | nd | 3.34E-06 | nd | 9.44E-07 | nd |
| NALM-6 | 2.15E-04 | 9.65E-02 | **449.02**^b^ | 1.67E-01 | **777.27** | 4.42E-02 | **205.38** |
| Rajy | 3.25E-04 | 1.25E-01 | **383.05** | 1.21E-01 | **372.28** | 4.42E-02 | **136.13** |
| U-937 | 8.54E-06 | **3.08E-04** | nd | **4.09E-04** | nd | 7.08E-05 | nd |
| KG-1a | 2.35E-06 | **5.77E-04** | nd | **5.82E-04** | nd | 6.79E-05 | nd |
| JURKAT | 1.70E-06 | **2.78E-04** | nd | **5.26E-04** | nd | 8.60E-06 | nd |
| K562 | 5.80E-07 | 2.56E-06 | nd | 1.63E-05 | nd | 1.42E-06 | nd |
| Ri-1 | 8.14E-05 | **1.29E-01** | nd | **1.11E-01** | nd | **4.58E-02** | nd |
| JY | 1.26E-05 | **3.33E-02** | nd | **4.21E-02** | nd | **3.18E-03** | nd |
| Mean | 6.49E-05^c^ | 3.84E-02 |  | 4.43E-02 |  | 1.38E-02 |  |
| SD | 1.14E-04 | 5.55E-02 |  | 6.41E-02 |  | 2.14E-02 |  |
| Dunn Test  *vs.* CTRL |  | *p*=0.0002 |  | *p<*0.0001 |  | *p*=0.2498 |  |

^a^, In bold gene expression induced (CXCL9/β-actin molecules ≥1E-04) in constitutively CXCL9-negative cells; ^b^, in bold gene expression up-regulated (FC≥2) in constitutively CXCL9-positive cells; ^c^, mean value of all investigated cell lines; nd, not detected.

**Supplementary Table 21. qRT-PCR analysis of MICB expression in DHAs-treated hematological cancer cell lines.**

| Hematological  tumor cell lines | CTRL | Guadecitabine | FC *vs.*  CTRL | DAC | FC *vs.*  CTRL | AZA | FC *vs.*  CTRL |
| --- | --- | --- | --- | --- | --- | --- | --- |
| Daudi | 1.71E-02 | 1.01E-02 | 0.59 | 1.57E-02 | 0.92 | 8.04E-03 | 0.47 |
| HL-60 | 6.90E-03 | 6.11E-03 | 0.88 | 7.30E-03 | 1.06 | 5.09E-03 | 0.74 |
| NALM-6 | 1.13E-02 | 1.11E-02 | 0.98 | 1.27E-02 | 1.13 | 4.87E-03 | 0.43 |
| Rajy | 9.25E-03 | 1.21E-02 | 1.31 | 1.16E-02 | 1.26 | 4.34E-03 | 0.47 |
| U-937 | 1.72E-02 | 1.07E-02 | 0.62 | 1.28E-02 | 0.74 | 1.08E-02 | 0.63 |
| KG-1a | 1.24E-02 | 1.17E-02 | 0.95 | 1.42E-02 | 1.15 | 1.29E-02 | 1.05 |
| JURKAT | 1.51E-02 | 1.21E-02 | 0.80 | 1.27E-02 | 0.84 | 1.20E-02 | 0.79 |
| K562 | 3.94E-03 | 1.59E-02 | **4.02**^a^ | 1.67E-02 | **4.22** | 1.60E-02 | **4.06** |
| Ri-1 | 1.70E-02 | 9.86E-03 | 0.58 | 9.69E-03 | 0.57 | 4.57E-03 | 0.27 |
| JY | 9.35E-03 | 1.24E-02 | 1.33 | 1.20E-02 | 1.28 | 8.81E-03 | 0.94 |
| Mean | 1.19E-02^b^ | 1.12E-02 |  | 1.25E-02 |  | 8.75E-03 |  |
| SD | 4.64E-03 | 2.45E-03 |  | 2.72E-03 |  | 4.10E-03 |  |
| Dunn Test  *vs.* CTRL |  | *p*=0.8961 |  | *p>*0.9999 |  | *p*=0.0459 |  |

^a^, In bold gene expression up-regulated (FC≥2) in constitutively MICB-positive cells; ^b^, mean value of all investigated cell lines.

**Supplementary Table 22. qRT-PCR analysis of MICA expression in DHAs-treated hematological cancer cell lines.**

| Hematological  tumor cell lines | CTRL | Guadecitabine | FC *vs.*  CTRL | DAC | FC *vs.*  CTRL | AZA | FC *vs.*  CTRL |
| --- | --- | --- | --- | --- | --- | --- | --- |
| Daudi | 1.36E-03 | 9.27E-04 | 0.68 | 7.09E-04 | 0.52 | 5.69E-04 | 0.42 |
| HL-60 | 7.27E-04 | 5.99E-04 | 0.82 | 7.13E-04 | 0.98 | 4.42E-04 | 0.61 |
| NALM-6 | 1.46E-03 | 5.75E-04 | 0.39 | 1.08E-03 | 0.74 | 3.61E-04 | 0.25 |
| Rajy | 1.23E-03 | 8.09E-04 | 0.66 | 8.45E-04 | 0.69 | 2.92E-04 | 0.24 |
| U-937 | 4.55E-03 | 2.68E-03 | 0.59 | 3.85E-03 | 0.85 | 1.50E-03 | 0.33 |
| KG-1a | 3.87E-03 | 2.92E-03 | 0.75 | 3.44E-03 | 0.89 | 1.53E-03 | 0.39 |
| JURKAT | 1.87E-03 | 2.34E-03 | 1.25 | 3.63E-03 | 1.94 | 1.27E-03 | 0.68 |
| K562 | 3.35E-03 | 7.59E-03 | **2.27**^a^ | 1.20E-02 | **3.58** | 6.98E-03 | **2.09** |
| Ri-1 | 1.39E-03 | 7.55E-04 | 0.54 | 7.02E-04 | 0.51 | 3.36E-04 | 0.24 |
| JY | 1.66E-03 | 1.76E-03 | 1.06 | 1.83E-03 | 1.10 | 1.00E-03 | 0.60 |
| Mean | 2.15E-03^b^ | 2.10E-03 |  | 2.88E-03 |  | 1.43E-03 |  |
| SD | 1.29E-03 | 2.13E-03 |  | 3.45E-03 |  | 2.01E-03 |  |
| Dunn Test  *vs.* CTRL |  | *p*=0.0002 |  | *p<*0.0001 |  | *p*=0.113 |  |

^a^, In bold gene expression up-regulated (FC≥2) in constitutively MICA-positive cells; ^b^, mean value of all investigated cell lines.
